# Identification of a highly expressed gene cluster likely coding for benzene activation enzymes in a methanogenic enrichment culture

**DOI:** 10.1128/aem.02083-25

**Published:** 2026-03-27

**Authors:** Courtney R. A. Toth, Olivia Molenda, Camilla L. Nesbø, Fei Luo, Cheryl E. Devine, Xu Chen, Kan Wu, Johnny Z. Xiao, Rishika Puri, Shen Guo, Nancy Bawa, Po-Hsiang Wang, Yifeng Wei, Robert Flick, Elizabeth A. Edwards

**Affiliations:** 1 Department of Chemical Engineering and Applied Chemistry, University of Toronto 686119, Toronto, Ontario, Canada; 2 Singapore Institute of Food and Biotechnology Innovation, Agency for Science, Technology and Research (A*STAR), Singapore, Singapore; University of Nebraska-Lincoln, Lincoln, Nebraska, USA

**Keywords:** benzene, *Desulfobacterota*, ORM2, “*Candidatus *Anaerobenzenivorax”, proteomics, bioremediation, biodegradation, anaerobic hydrocarbon degradation, methanogenesis

## Abstract

The oil refinery (OR) consortium is a model methanogenic enrichment culture used to study anaerobic benzene degradation. Over half of the culture’s bacterial community consists of two closely related *Desulfobacterota* strains, designated ORM2a and ORM2b, whose mechanisms of benzene activation are unknown. Three proteomics data sets were integrated and analyzed using high-quality OR metagenomes and metagenome-assembled genomes (MAGs), including a complete circularized ORM2a MAG, to identify active metabolic pathways and proteins expressed during methanogenic benzene degradation. Among the proteins identified were Bam-like subunits of an ATP-independent benzoyl-CoA degradation pathway, as well as downstream β-oxidation proteins yielding acetate. The most abundant proteins identified mapped to two ORM2a gene clusters of unknown function. Homologous and syntenic gene clusters were identified in the MAGs of ORM2b and a sulfate-reducing *Pelotomaculum* that also degrades benzene, as well as in nine contigs assembled from hydrothermal vent metagenomes. Extensive homology and structural predictions suggest that the first cluster—termed the “Magic” gene cluster—encodes for enzymes catalyzing the chemically challenging activation of benzene and subsequent transformation steps yielding benzoyl-CoA. The second (“Nanopod”) gene cluster encodes a transmembrane complex that may facilitate benzene transport across the cell membrane. Phylogenomic analyses place ORM2a and ORM2b within a novel genus of strict anaerobes specialized for benzene degradation, which we propose naming “*Candidatus* Anaerobenzenivorax.”

**IMPORTANCE:**

Benzene is a widespread, persistent, and toxic pollutant that can accumulate in anoxic environments such as groundwater and sediments. Benzene can be metabolized in the absence of oxygen; however, despite decades of research, the biochemical mechanisms for benzene activation under anaerobic conditions remain unproven. This study provides strong genetic and proteomic evidence for a new suite of enzymes that initiate anaerobic benzene activation. These findings lay a foundation for future biochemical studies and expand our understanding of how microbes carry out difficult chemical reactions in the absence of oxygen.

## INTRODUCTION

It has been more than 45 years since anaerobic benzene degradation was first documented by Ward et al. (1), reversing a long-held tenet that oxygen was required for the degradation of aromatic hydrocarbons. Notorious for its carcinogenicity, stringent environmental regulations, and persistence in anoxic environments, benzene has been the subject of numerous studies seeking to characterize its metabolism in the absence of oxygen (2, 3). Laboratory enrichment culture studies have confirmed unequivocally that anaerobic benzene degradation can be coupled to iron reduction, nitrate reduction, sulfate reduction, and methanogenesis, catalyzed by a handful of specialized microbial clades (2, 4). Despite great efforts, the biochemical mechanism(s) by which benzene is activated in the absence of oxygen remain elusive.

Initial studies using ^13^C- or ^14^C-benzene and occasionally H_2_^18^O identified three possible activation reactions based on the detection of labeled metabolites: (i) anaerobic hydroxylation of benzene yielding phenol (5–12), (ii) carboxylation of benzene yielding benzoate (6, 8–10, 13, 14), and (iii) methylation of benzene yielding toluene (10). However, neither phenol nor benzoate is diagnostic of a single activation mechanism (15), and supporting evidence of benzene methylation is sparse in the literature (16, 17). From 2003 to 2008, hydrogen and carbon isotope fractionation ratios were evaluated in active benzene-degrading enrichment cultures (18–20). The slope of dual isotope plots (∆δ ^2^H/∆δ ^13^C, a value defined as Λ) from nitrate-reducing cultures (Λ = 8–19, *n* = 5) was substantially lower than for sulfate-reducing and methanogenic cultures surveyed (Λ = 28–31, *n* = 7), hinting that at least two distinct mechanisms of anaerobic benzene activation exist in nature (18).

By 2010, advances in DNA sequencing and analysis enabled meta-omics characterization of anaerobic benzene degraders for the first time (21–23). Abu Laban et al. (24) sequenced the metagenome of the iron-reducing enrichment culture BF (“benzene-ferrihydrite”) and identified a protein with homology to a known phenylphosphate carboxylase (PpcA). The corresponding putative anaerobic benzene carboxylation gene (*abcA*) was located on a contig affiliated with *Clostridia*. Expression of the putative *abcA* and neighboring genes (*abcD*, benzoyl-CoA ligase [*bzlA*], and a *ubiX*-like gene) was induced with benzene and benzoate, but not with phenol, suggesting a possible role in benzene activation (24). Expression of syntenic gene clusters has since been detected in the transcriptomes of several nitrate-reducing cultures (25–27), including one previously analyzed by isotopic fractionation (18, 19). Most nitrate- and iron-reducing benzene degraders identified to date have been affiliated with the order *Thermincolales* (25–29); a putative benzene-carboxylating, sulfate-reducing *Thermincolales* was also recently identified in a jet fuel-contaminated aquifer via metagenomics (30). Researchers are now attempting to verify benzene carboxylation using structural and biochemical assays, among other tools (31).

Evidence for benzene hydroxylation has only been shown for one microorganism, *Geobacter metallireducens* GS-15, via gene deletion. In 2013–2014, Zhang et al. (5, 23) identified a handful of genes that were upregulated during active phenol or benzene metabolism, and deletion of key genes (Gmet0231, Gmet0232, or *ppsA*) inhibited benzene degradation. The *ppsA* gene encodes for the alpha subunit of phenylphosphate synthase, which catalyzes the first step in anaerobic phenol metabolism (32, 33). Functional annotations suggest that the products of Gmet0231 and Gmet0232 encode oxidoreductases that could potentially catalyze the addition of water to benzene, forming phenol. Further investigation into the exact functions of these enzymes is needed. In 2024, Bullows et al. (17) identified a fumarate addition gene cluster consisting of a 3-hydroxybenzylsuccinate synthase and a methyltransferase in the genome of *Geotalea daltonii* strain FRC-32, a close relative of *Geobacter*. Expression of these genes, previously known only to catalyze the activation of alkanes and alkyl-substituted aromatic rings (34), was upregulated during active benzene metabolism but not with toluene. Addition of benzene to *G. daltonii* lysates resulted in near-stoichiometric formation of toluene, but curiously, benzene was nearly completely depleted before toluene was formed (17). Whether this gene cluster truly encodes for benzene methylation enzymes remains uncertain.

Metagenomic and proteomic approaches have also been applied to sulfate-reducing and methanogenic benzene-degrading enrichment cultures. To date, these surveys have consistently identified only the presence and expression of genes implicated in the ATP-independent metabolism of benzoyl-CoA and downstream β-oxidation intermediates (21, 35). Homologs of known and predicted anaerobic aromatic-activating genes have not been detected in methanogenic benzene-degrading enrichment cultures (4, 21) or in the sulfate-reducing benzene-degrading enrichment culture BPL (9, 35). Likewise, genes associated with aerobic benzene metabolism, such as monooxygenases, have not been detected in these cultures, although recent oxygen exposure experiments indicated that aerobic benzene degraders may persist at trace abundances (36). Additionally, these enrichments lack genes encoding benzoyl-CoA ligase and do not exhibit growth on benzoate (21, 35). Together with isotope fractionation data (18, 19) indicating that benzene activation in methanogenic cultures proceeds via a mechanism distinct from nitrate-reducing systems—where *abcA* genes are present, highly expressed, and carboxylation is likely (25–27)—we surmise that a novel hydrocarbon activation mechanism catalyzing anaerobic transformation of benzene to benzoyl-CoA must exist in methanogenic systems.

In this study, we integrated the results from three proteomic experiments on the methanogenic benzene-degrading OR consortium, a highly enriched culture that has been maintained on benzene as its sole carbon and energy source for over 25 years (37, 38). For the reader’s convenience, a research overview of the OR consortium and key findings relevant to this study are provided in Fig. S1 and Table S1, while representative benzene biodegradation trends and microbial community profiles are shown in Fig. S2 and Table S2. The OR consortium harbors two closely related strains of *Desulfobacterota* (ORM2a and ORM2b) that are consistently detected in high abundance (4, 21, 36, 37, 39–42). Although the relative abundance of each ORM2 strain varies by subculture (40), together, they account for 48%–92% of total bacteria (Table S2). The culture also contains methanogenic archaea (*Methanothrix* and *Methanoregula*) and over 100 low-abundance microorganisms with various predicted functions (40, 41, 43). Using quantitative PCR and growth yield estimates, we have repeatedly demonstrated that ORM2a and ORM2b are the only consortium members whose growth directly coincides with methanogenic benzene degradation (4, 21, 36, 40). Neither strain has been isolated in pure culture, and no substrate other than benzene has been shown to support their growth (10, 21, 40).

Initial metagenomic and proteomic surveys of the methanogenic OR consortium were conducted in the early 2010s but were not published beyond a PhD thesis (21) due to challenges in data interpretation and low protein yields. Subsequent improvements in culture maintenance (36, 40) and sequencing efforts enabled the reconstruction of two high-quality metagenomes and 74 metagenome-assembled genomes (MAGs; Table S3), including a complete circular metagenome-assembled genome (cMAG) for ORM2a (NCBI accession no. CP113000.1) that was peer-reviewed and published in Microbial Resource Announcements (41). In the present study, these existing resources were reanalyzed alongside a newer, higher-quality proteomics data set using updated bioinformatic approaches, enabling a substantially more refined interpretation of benzene metabolism in the OR consortium. Notably, the most abundant proteins expressed by the culture mapped to the ORM2a genome, specifically to a unique cluster of syntenic genes found only in other benzene-degrading obligate anaerobes and in a limited number of metagenomic contigs from hydrocarbon-producing hydrothermal vents.

## RESULTS

### Features of the ORM2a cMAG

**Fig 1:**
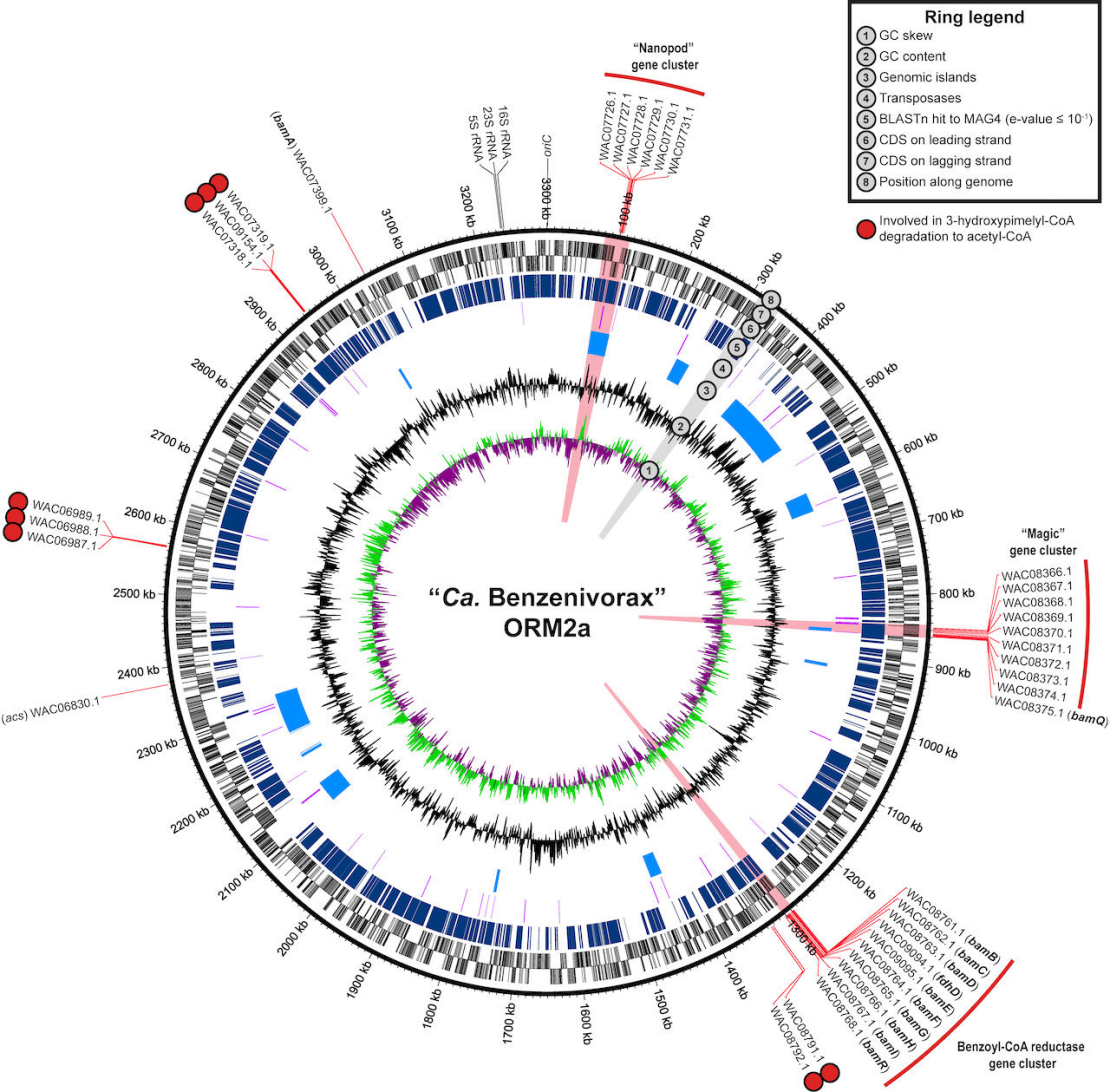
The complete ORM2a genome. Genomic neighborhoods and genes of interest, whose products were recovered with high certainty (≥99% confidence) from the OR proteomes, are highlighted in red and summarized in Table 2. Genes involved in the degradation of 3-hydroxypimelyl-CoA to acetyl-CoA are marked with red circles. Protein accession numbers are provided, and corresponding ORM2a gene locus tags are listed in Table S11. The positions of the origin of replication (*oriC*) and ribosomal RNA genes are also shown in this figure.

**Fig 2:**
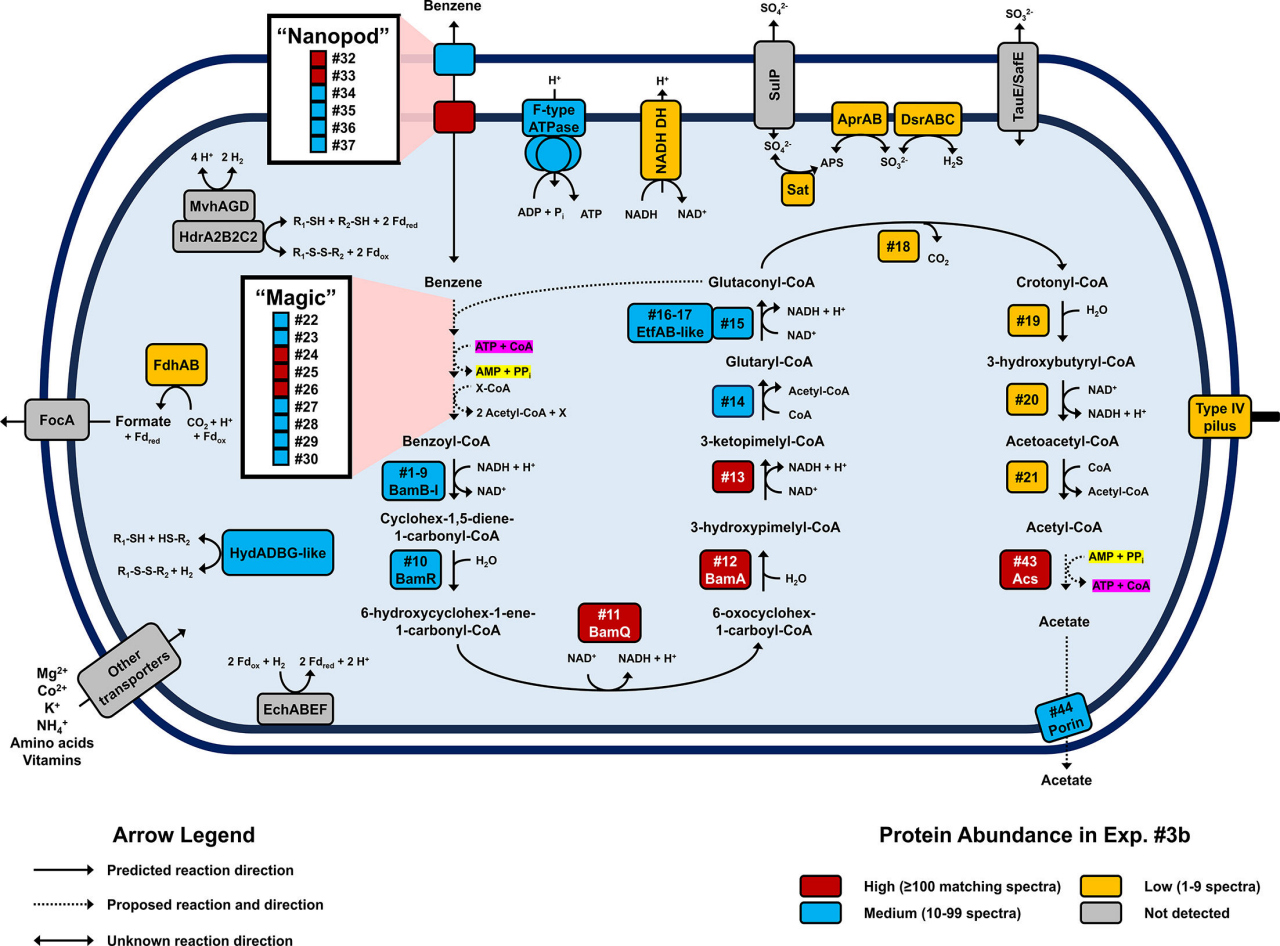
Summary of key metabolic pathways identified in the ORM2a proteome. Protein spectral counts from Experiment #3b were used to estimate protein abundances. Additional complete and incomplete metabolic pathways identified in the ORM2a genome are summarized in Table S5. Annotations for numbered proteins are provided in Table 2. Abbreviations (clockwise from top-center): ATPase, ATP synthase; NADH DH; NADH dehydrogenase; SulP, sulfate permease; Sat, sulfate adenylyltransferase; AprAB, adenylyl-sulfate reductase; Dsr, dissimilatory sulfite reductase; TauE/SafE, sulfite exporter; Acs, acetyl-CoA synthetase; EchABEF, energy-converting hydrogenase; HydABGD-like, sulfhydrogenase; R-S-S-R, heterodisulfide; FocA, formate-nitrite transporter; FdhAB, formate dehydrogenase; Fd, ferredoxin; HdrA2B2C2, F420-reducing heterodisulfide reductase; and Mvh, F420-non-reducing hydrogenase.

The complete, closed metagenome-assembled genome of ORM2a consists of a single circular chromosome of 3,300,546 bp with low GC skew, no discernible terminus region (Fig. 1; Fig. S3), and a high mean read depth of 992.7 ± 112.4× (41). General genome statistics are summarized in Table S4. To assess metabolic potential, coding sequences predicted by the NCBI Prokaryotic Genome Annotation Pipeline were uploaded to BlastKOALA for automatic KEGG Orthology (KO) assignment (44). Forty-six percent of genes identified (1,431 of 3,135) were assigned KO identifiers and mapped into metabolic pathways using the KEGG Mapper Reconstruct tool (45, 46). Key pathways and genes of interest are highlighted below and in Fig. 2; additional pathway and genomic features are detailed in Tables S5 to S10 and Text S1.

The ORM2a cMAG contains few and mostly incomplete pathways for metabolizing carbohydrates, lipids, amino acids, and nucleic acids. Similarly, pathways for electron transport and ATP generation are sparse, comprising an F-type ATPase, a Nuo-type NADH dehydrogenase with one missing subunit (*nuoG*), and a complete dissimilatory sulfate reduction pathway (Fig. 2). This finding supports recent experimental evidence that the OR consortium can couple benzene oxidation to sulfate reduction in addition to methanogenesis (4). BLAST searches and manual inspections confirmed the absence of homologs to known hydrocarbon-activating genes and benzoyl-CoA ligase. The latter is a significant distinction between microorganisms containing putative anaerobic benzene carboxylase genes like *abcA* and those that do not (35). The presence of a complete ATP-independent benzoyl-CoA degradation pathway is consistent with ORM2a’s involvement in anaerobic benzene oxidation (Fig. 1 and 2).

Additional genomic evidence suggests that ORM2a encodes multiple hydrogenase systems (Fig. 2; Table S7). Three gene clusters encoding energy-converting hydrogenase (Ech) and formate dehydrogenase (Fdh) complexes were identified that could support a syntrophic lifestyle by exporting H_2_ and formate to partner methanogens (47, 48). The cMAG also encodes a cytoplasmic Mvh-type F_420_-non-reducing hydrogenase, which in other organisms is implicated in hydrogen uptake during autotrophic growth on CO_2_ (49, 50). ORM2a also harbors a gene subcluster putatively encoding a sulfohydrogenase. These poorly characterized enzymes typically reduce elemental sulfur (S⁰) or polysulfides (S_n_^2-^) to hydrogen sulfide (H_2_S) using H_2_ as an electron donor (51). This process has been most thoroughly described in *Pyrococcus furiosus* and related thermophilic sulfur-oxidizing archaea (51–54). Under certain conditions, some sulfhydrogenases may also catalyze the reverse reaction, producing H_2_ (51, 53). The reaction direction catalyzed in ORM2a is unknown, as are its sulfur substrates. The only major sulfur source in the OR growth medium is ferrous sulfide (FeS), added as a reductant (37). There is no published evidence that sulfhydrogenases can oxidize FeS or reduce oxidized forms such as FeS_2_. Furthermore, no visible S^0^ or polysulfide formation (e.g., yellow solids) has ever been observed in the >25 years this culture has been maintained.

A draft MAG belonging to ORM2b (designated ORM2b_MAG4) was assembled into 378 contigs and is estimated to be 74.6% complete (41). Although the exact taxonomic identity of ORM2b_MAG4 remains uncertain due to the absence of a 16S rRNA gene—precluding direct comparison to a reference clone sequence of ORM2b (NCBI accession no. KT025834)—the high average nucleotide identity (89.94%) between the ORM2a cMAG and ORM2b_MAG4 supports its correct classification (Fig. 1, ring 5; Fig. S4). No other MAGs recovered from the OR consortium metagenomes display significant nucleotide identity to ORM2a.

### Proteomic analyses

Between 2010 and 2018, three proteomics experiments were performed on subcultures of the OR consortium (OR-b1C, OR-b, and OR-b1A; Fig. S1). Experiments #1 (2010) and #2 (2011) were conducted by Devine (21) and used LC-MS/MS to analyze trypsin-digested crude extracts. We reprocessed her spectral data sets against a newer protein discovery library (Table S3; File S1) but could only identify 153 unique proteins (Table 1)—a very low yield. To improve detection, protein separation by gel electrophoresis was introduced in Experiment #3 (2018). Duplicate crude extracts were prepared: one was directly digested with trypsin (Experiment #3a), as in previous protocols, and yielded similarly few proteins (Table 1). The second crude extract (Experiment #3b) was separated on a 15% SDS-PAGE gel, sliced into five equal sections (Fig. S5), and subjected to in-gel trypsin digestion and LC-MS/MS. This method yielded 825 unique proteins (Table 1).

**TABLE 1:** Summary of proteomic experiments performed on the oil refinery enrichment culture^*a*^

Experiment no. (year)	OR subculture used	Benzene degradation rate (mg/L/day)	Culture volume extracted (mL)	Protein purification methodology	Protein separation methodology	Total no. of spectra detected	No. of unique proteins identified	No. of unique proteins assigned to designated MAG(s) (% of unique proteins identified)				
								ORM2a	MAG4_ ORM2b	Both ORM2a and MAG4_ ORM2b	Archaeal MAGs	Other bacterial MAGs
1 (2010)	OR-b1C	0.3	175	Phenol-methanol/ammonium acetate extraction	None	701	119	29 (24%)	14 (12%)	0 (0%)	37 (31%)	2 (2%)
2a (2011)	OR-1b	0.4	175	Phenol-methanol/ammonium acetate extraction	None	228	49	20 (41%)	3 (6%)	1 (2%)	13 (27%)	1 (2%)
2b (2011)	OR-1b	0.4	175	Phenol-methanol/ammonium acetate extraction	None	457	74	27 (36%)	5 (7%)	0 (0%)	21 (28%)	1 (1%)
3a (2018)	OR-b1A	0.7	50	Filter-aided sample preparation	None	47	24	12 (50%)	0 (0%)	1 (4%)	6 (25%)	2 (8%)
3b (2018)	OR-b1A	0.7	50	Filter-aided sample preparation	15% SDS-PAGE	10,914	825	347 (42%)	25 (3%)	44 (5%)	185 (22%)	50 (6%)
					**Average**			**39%**	**6%**	**2%**	**27%**	**4%**
					**Total no. of unique proteins identified**			**348**	**33**	**44**	**189**	**50**

^*a*^ Experiments differed in sample preparation methods, fractionation approaches, and instrumentation. Experiment #1 analyzed one protein sample, while Experiments #2 and #3 included technical replicates (a and b). All samples were subjected to protein extraction by sonication.

**Fig 3:**
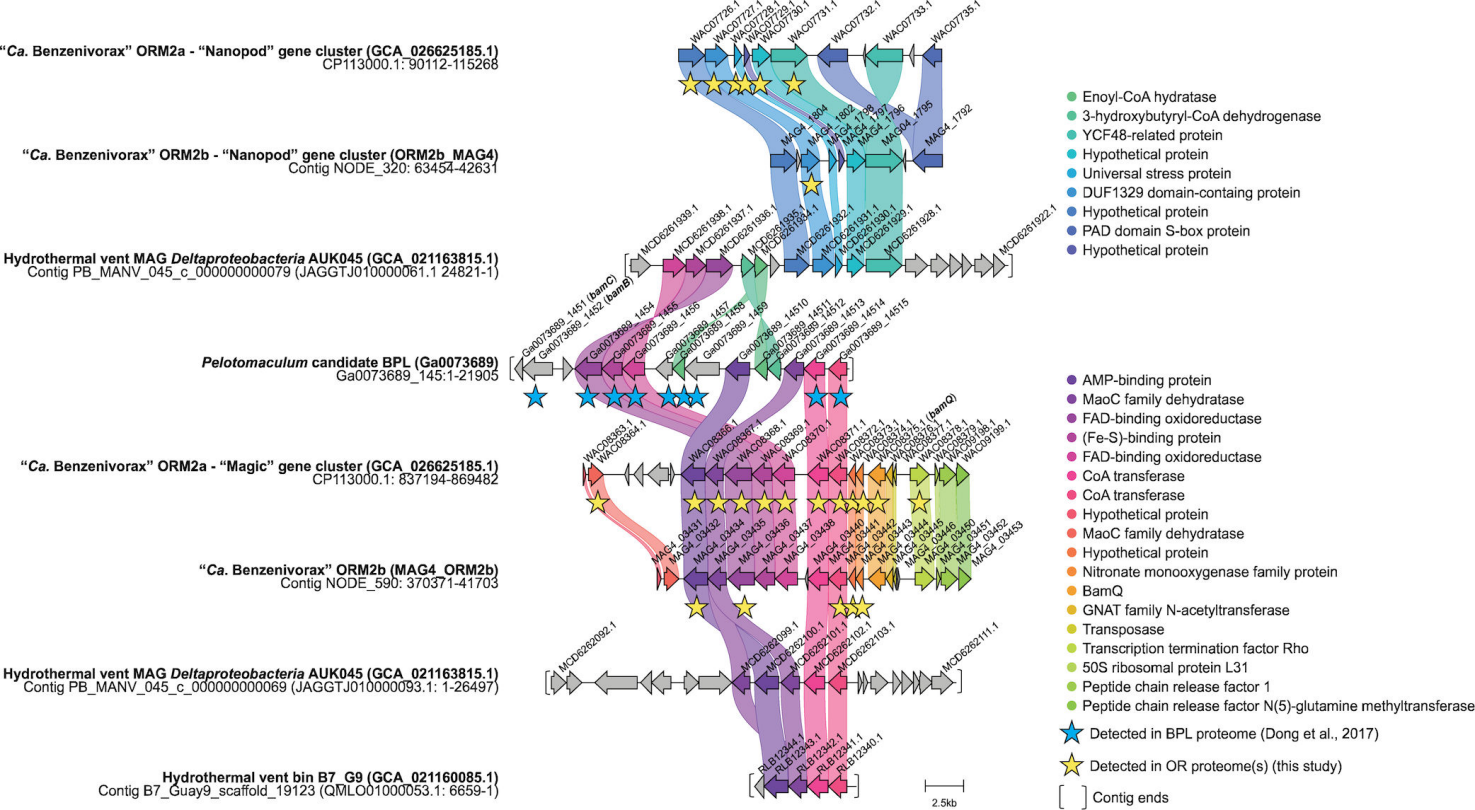
Genomic neighborhoods of all publicly available MAGs in NCBI and IMG producing significant alignments to the “Magic” and “Nanopod” gene clusters in ORM2a and ORM2b_MAG4. Homologous genes (≥40% amino acid identity) are connected and color-coded by function; sequence alignment statistics for relevant proteins are shown in Table 3, and additional homology statistics are provided in Table S14. Corresponding products detected in the proteomes of the OR consortium and the enrichment culture BPL are marked with yellow or blue stars, respectively. The ends of a contig are capped with brackets.

**Fig 4:**
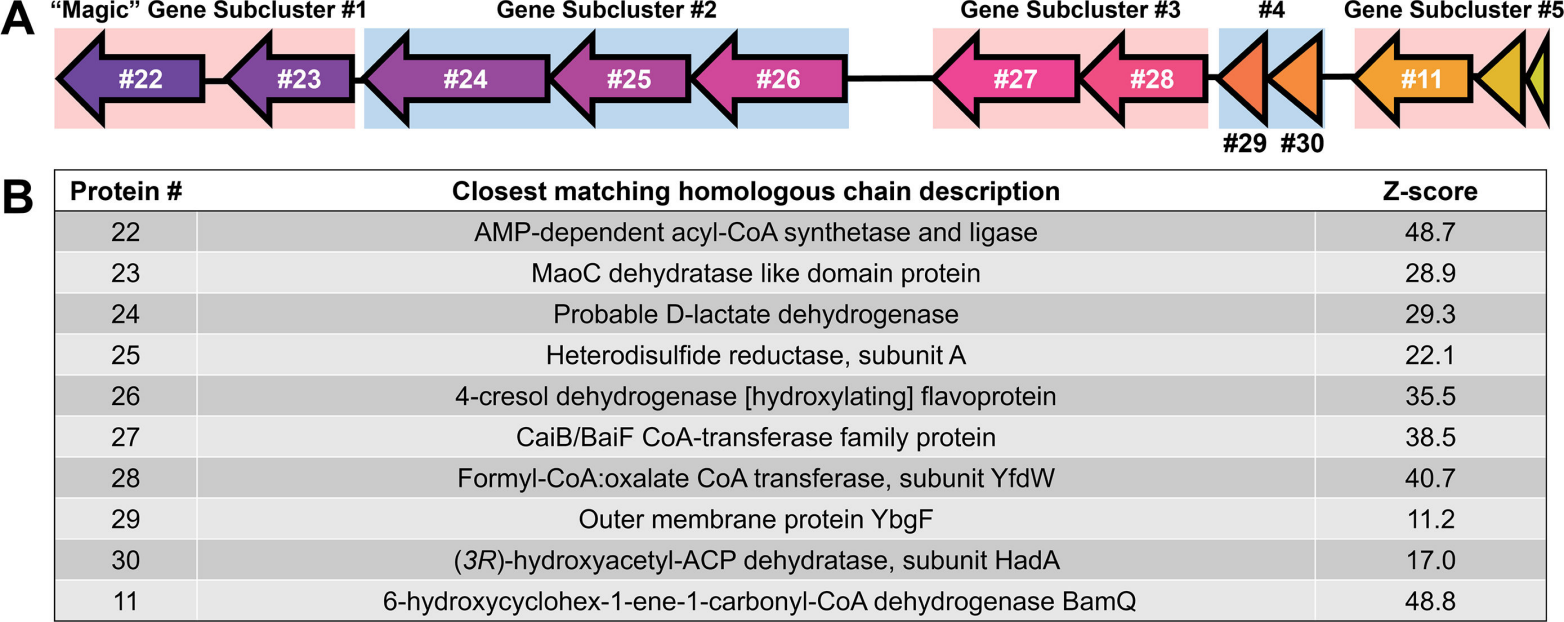
Structure and functional prediction of proteins encoded by the “Magic” gene cluster. Panel **A** shows the organization of the gene cluster and its predicted subclusters. Panel **B** summarizes the best Protein Data Bank (PDB) search results for each “Magic” gene product. Z-scores are a measure of protein composite confidence, and Z-scores ≥30 are considered significant. Additional alignment statistics are provided in Table S15.

**Fig 5:**
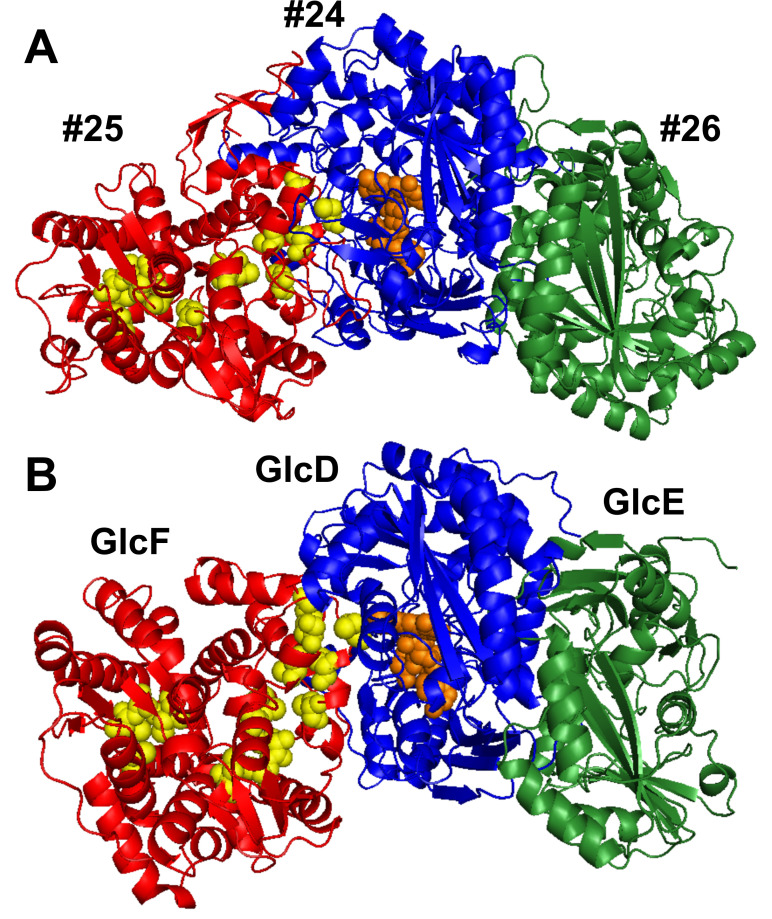
AlphaFold 3 predicted model of (**A**) the Protein #24–26 enzyme complex and (**B**) the glycolate dehydrogenase complex GlcDEF in *Escherichia coli*. Corresponding subunits are shown in red, blue, or green. FAD is rendered as orange spheres, and conserved cysteine residues potentially binding [4Fe–4S] clusters are shown as yellow spheres. A putative binding pocket is located near the FAD-binding site in Protein #24, which can be seen in Fig. S9A. Structure confidence scores for the Protein #24–26 complex are shown in Fig. S9B. Protein structure alignment and superposition of both complexes are provided in Fig. S10.

In total, 858 unique proteins were identified across all experiments (Table 1). Protein accession numbers, automated annotations, peptide and spectral counts, and amino acid sequences are provided in Table S11, with summary statistics in Table S12a and b. Proteins that are putatively involved in anaerobic benzene degradation or were highly detected in Experiment #3b (≥100 spectra) are highlighted in Table 2 and Fig. 1 to 5. Notably, the most highly detected proteins in Experiment #3b were also recovered in Experiments #1 and #2 (Table S11), demonstrating strong reproducibility of our results despite years between tests and differences in extraction and analysis methods (Table 1).

**TABLE 2:** Summary of highly detected proteins (≥100 matching spectra in Experiment #3b) and proteins of interest identified with ≥99% confidence^*a*^

Protein no.	Predicted function (NCBI prokaryotic genome annotation pipeline version 6.3 and best BLASTP hits)	Total no. of spectra detected (Exp. #3b)	Protein abundance rank	Matching MAG(s)	Protein accession no.
*Benzoyl-CoA degradation to 3-hydroxypimelyl-CoA*					
1	Benzoyl-CoA reductase subunit BamB	80	=26	ORM2a and ORM2b	WAC08761.1, ORM2b_MAG4_01005
2	Benzoyl-CoA reductase subunit BamC	15	=154	ORM2a and ORM2b	WAC08762.1, ORM2b_MAG4_01004
3	Benzoyl-CoA reductase subunit BamD	61	=43	ORM2a	WAC08763.1
4	Formate dehydrogenase accessory sulfurtransferase FdhD	6	=287	ORM2a	WAC09094.1
5	Benzoyl-CoA reductase subunit BamE	68	32	ORM2a	WAC09095.1
6	Benzoyl-CoA reductase subunit BamF	18	=135	ORM2a	WAC08764.1
7	Benzoyl-CoA reductase subunit BamG	6	=287	ORM2a	WAC08765.1
8	Benzoyl-CoA reductase subunit BamH	91	23	ORM2a and ORM2b	WAC08766.1, ORM2b_MAG4_04225
9	Benzoyl-CoA reductase subunit BamI	9	=227	ORM2a	WAC08767.1
10	Cyclohexa-1,5-diene-1-carbonyl-CoA hydratase BamR	49	59	ORM2a	WAC08768.1
**11**	**6-hydroxycyclohex-1-ene-1-carbonyl-CoA dehydrogenase BamQ**	**103**	**20**	**ORM2a**	**WAC08375.1**
**12**	**6-oxo-cyclohex-1-ene-carbonyl-CoA hydrolase BamA**	**143**	**13**	**ORM2a**	**WAC07399.1**
*3-hydroxypimelyl-CoA degradation to acetyl-CoA*					
**13**	**3-hydroxyacyl-CoA dehydrogenase**	**148**	**9**	**ORM2a**	**WAC08792.1**
14	Thiolase family protein	69	31	ORM2a	WAC08791.1
15	Glutaryl-CoA dehydrogenase (non-decarboxylating)	58	=49	ORM2a	WAC06987.1
16	Electron transfer flavoprotein subunit beta/FixA family protein	39	=69	ORM2a	WAC06988.1
17	Electron transfer flavoprotein subunit alpha/FixB family protein	63	=39	ORM2a	WAC06989.1
18	Glutaconyl-CoA decarboxylase subunit alpha	5	=332	ORM2a	WAC08228.1
19	Enoyl-CoA hydratase-related protein	3	=458	ORM2a	WAC07318.1
20	3-hydroxybutyryl-CoA dehydrogenase	9	=227	ORM2a	WAC09154.1
21	Acetyl-CoA C-acetyltransferase	3	=458	ORM2a	WAC07319.1
*Highly expressed “Magic” gene cluster putatively involved in benzene activation*					
22	AMP-binding protein	74	29	ORM2a and ORM2b	WAC08366.1, ORM2b_MAG4_03434
23	MaoC family dehydratase	57	52	ORM2a	WAC08367.1
**24**	**FAD-binding oxidoreductase**	**172**	**3**	**ORM2a**	**WAC08368.1**
**25**	(**Fe-S)-binding protein**	**219**	**2**	**ORM2a**	**WAC08369.1**
**26**	**FAD-binding oxidoreductase**	**265**	**1**	**ORM2a**	**WAC08370.1**
27	Type III CoA transferase	45	61	ORM2a	WAC08371.1
28	Type III CoA transferase	58	=49	ORM2a and ORM2b	WAC08372.1, ORM2b_MAG4_03441
29	Hypothetical protein	19	=128	ORM2a and ORM2b	WAC08373.1, ORM2b_MAG4_03442
30	MaoC family dehydratase	50	=57	ORM2a	WAC08374.1
31	Transcription termination factor Rho	25	=100	ORM2a	WAC08378.1
*Highly expressed “Nanopod” gene cluster putatively involved in benzene transportation across cell membrane*					
**32**	**Hypothetical protein**	**106**	**=18**	**ORM2a**	**WAC07726.1**
**33**	**DUF1329 domain-containing protein**	**158**	**4**	**ORM2a**	**WAC07727.1, ORM2b_MAG4_01802**
34	Universal stress protein	6	=287	ORM2a	WAC07728.1
35	Hypothetical protein	7	=269	ORM2a	WAC07729.1
36	YCF48-related protein	65	=36	ORM2a	WAC07730.1
37	MMPL family transporter	60	=45	ORM2a	WAC07731.1
*Archaeal proteins associated with methanogenesis*					
**38**	**Acetate-CoA synthetase Acs**	**142**	**14**	***Methanothrix* MAG20**	**MAG20_01236**
**39**	**Coenzyme-B sulfoethylthiotransferase McrB**	**149**	**8**	***Methanothrix* MAG21**	**MAG21_01157**
**40**	**Coenzyme-B sulfoethylthiotransferase McrB**	**154**	**7**	***Methanothrix* MAG11, MAG20 and MAG28**	**MAG11_02361, MAG20_02648, MAG28_00841**
**41**	**Acetate-CoA synthetase Acs**	**125**	**16**	***Methanothrix***	**MAG11_00500**
*Other proteins*					
**42**	**Chaperonin GroL**	**156**	**=5**	**ORM2a**	**WAC08671.1**
**43**	**Acetate-CoA synthetase Acs**	**156**	**=5**	**ORM2a**	**WAC06830.1**
44	Porin	53	53	ORM2a	WAC06831.1

^*a*^ Proteins shown are mapped to one or more MAGs reconstructed from OR metagenomes. Proteins are grouped by predicted biochemical functions and ranked by total spectrum counts, with ties indicated by equal symbols (=). Additional proteins identified are listed in Table S11. Highly detected proteins are highlighted in bold.

### Key microorganisms identified in the OR proteomes

Half of all proteins identified mapped to the cMAG of ORM2a and/or ORM2b_MAG4 (Tables 1 and 2). Of these, 348 proteins mapped exclusively to ORM2a (41%), 33 proteins mapped exclusively to ORM2b_MAG4 (4%), and 44 proteins (5%) mapped to both MAGs (Table 1). Most ORM2b_MAG4 proteins identified shared 71%–100% amino acid identity to a homologous ORM2a protein, while the remainder matched to undetected ORM2a gene products or were unique to ORM2b_MAG4. Only six proteins detected in low abundance—associated with protein synthesis or hypothetical functions—lacked ORM2a counterparts (Table S11). Given the predominance of ORM2a proteins detected in this study, the following sections focus on ORM2a proteins and metabolic pathways of interest.

One-quarter of all identified proteins mapped to MAGs of methanogenic archaea (Table 1; Table S10). The majority of these archaeal proteins were affiliated with acetoclastic *Methanothrix* spp. (170 proteins across 8 MAGs), whereas far fewer mapped to hydrogenotrophic *Methanoregula* spp*.* (19 proteins across 3 MAGs). Most archaeal proteins detected at high abundance were affiliated with methanogenesis and mapped to more than one MAG (Table 2). Forty-four proteins (5%) detected at low abundance mapped to other bacterial MAGs, including “*Ca*. Nealsonbacteria” DGGOD1a (42). The remaining proteins could not be assigned to a specific MAG (180 proteins, 21%) or mapped to contaminant/decoy sequences (20 proteins, 2%).

### Benzoyl-CoA degradation to acetyl-CoA

Across the OR metagenomes, we identified 146 predicted protein-coding genes associated with the metabolism of benzoyl-CoA and related aromatic intermediates, in the ORM2a and ORM2b MAGs, as well as in many low-abundance MAGs and unbinned contigs with low read depth (≤71×; Table S13). We searched the proteomic data and found corresponding proteins mapping only to the ORM2 MAGs. Specifically, we detected a complete suite of putative benzoyl-CoA degradation proteins encoded in the ORM2a cMAG (Fig. 1 and 2; Table 2). Ten gene products (designated Proteins #1–10) mapped to a single gene cluster and were annotated as subunits of a Bam-type benzoyl-CoA reductase (BamBCDEFDHI; whose components are described in Wischgoll et al. [55] and Boll et al. [56]), an FdhD chaperone, and a cyclohexa-1,5-diene-1-carbonyl-CoA hydratase (BamR). Two highly detected proteins annotated as 6-hydroxycyclohex-1-ene-1-carbonyl-CoA dehydrogenase (BamQ, Protein #11) and 6-oxo-cyclohex-1-ene-carbonyl-CoA hydrolase (BamA, Protein #12) were encoded elsewhere in the ORM2a cMAG (Fig. 1). Multiple sequence alignments (Table S14) and maximum likelihood phylogenetic tree analyses (Fig. S6) support the functional annotation of all predicted Bam proteins. Three *bam* gene products mapped to ORM2b_MAG4 (Table 2), but no additional benzoyl-CoA degradation proteins were detected. Together, these observations suggest that while the OR consortium harbors multiple organisms with the genetic potential to metabolize benzoyl-CoA, ORM2a and ORM2b are primarily responsible for benzoyl-CoA turnover during anaerobic benzene degradation (4, 21, 36, 40, 42).

Nine proteins predicted to catalyze the transformation of 3-hydroxypimelyl-CoA to acetyl-CoA were also identified and mapped exclusively to the ORM2a cMAG (Fig. 1 and 2; Table 2). In our most comprehensive proteome (Experiment #3b), proteins involved in converting 3-hydroxypimelyl-CoA to glutaconyl-CoA (Proteins #13–17) yielded more spectral counts than downstream β-oxidation enzymes (Proteins #18–21). While spectral counts provide only a semi-quantitative measure of protein abundance, the relative trend hints at a potential metabolic branch point at glutaconyl-CoA (Fig. 2), discussed later. Notably, the ORM2a cMAG contains a gene subcluster predicted to encode for a non-decarboxylating glutaryl-CoA dehydrogenase (GDH, Protein #15) and two electron-transferring flavoproteins (EtfAB, Proteins #16–17), which may serve as electron acceptors during the reduction of glutaryl-CoA to glutaconyl-CoA. Analogous electron-transferring reactions have been described for glutaryl-CoA dehydrogenase systems in both eukaryotes and bacteria, including *G. metallireducens* (57).

### Sulfur metabolism and acetate production

Several unexpected ORM2a proteins were detected in the OR proteomes, including six associated with the dissimilatory reduction of sulfate to hydrogen sulfide, specifically sulfate adenylyltransferase (Sat), adenylylsulfate reductase (AprAB), and dissimilatory sulfite reductase components (DsrAB and DsrC), despite not adding any sulfate to the culture medium (Fig. 2). Additionally, products of the putative sulfhydrogenase operon (HydADBG) were the only hydrogenase proteins detected that mapped to the cMAG of ORM2a (Fig. 2; Table S7). If this enzyme consumes H_2_ released during benzene oxidation, it could explain why so few proteins from hydrogenotrophic methanogens (*Methanoregula* spp.) were recovered from the OR proteomes (Table S11). Together, these findings raise new questions about ORM2a’s sulfur metabolism that warrant further investigation.

The protein folding chaperonin GroEL (Protein #42) and a putative AMP-forming acetyl-CoA synthetase (Acs, #43) were tied as the fifth most highly detected proteins in Experiment #3b (Table 2). While Acs typically activates acetate to acetyl-CoA, ORM2a already generates abundant acetyl-CoA during benzene metabolism. We therefore hypothesize that this Acs operates in reverse, converting acetyl-CoA and pyrophosphate (PP_i_) into acetate, ATP, and CoA (Fig. 2). This would simultaneously provide acetate to partner *Methanothrix* spp. and generate ATP for ORM2a. Similar reverse reactions were proposed for the anaerobic benzoate degrader *Syntrophus acidotrophicus* (58, 59) and three syntrophic bacteria whose MAGs were reconstructed from full-scale anaerobic digesters (60). None of these MAGs code for acetate kinase or closely related homologs needed to phosphorylate acetate. ORM2a also lacks cytosolic pyrophosphatase but encodes a membrane-bound Na^+^-translocating pyrophosphatase (WAC07798.1) that was detected in every proteome surveyed. This configuration may conserve intracellular PP_i_ levels and facilitate ATP production via the reverse Acs reaction (59). Phylogenetic trees support the correct annotation of Protein #43 (Fig. S7). An unclassified porin (Protein #44) whose corresponding gene is localized adjacent to *acs* may facilitate acetate export from ORM2a cells (Fig. 2).

### Highly expressed ORM2a proteins from gene clusters of unknown function

The three most consistent and highly detected proteins in all OR proteomes (Proteins #24–26) mapped consecutively to an ORM2a gene cluster of unknown function (Table 2; Fig. 1, 3, and 4A). Products of several neighboring genes (Proteins #22–23, #27–30) were also detected in multiple OR proteomes. This region, designated herein as the “Magic” gene cluster, is located on a small genomic island flanked by multiple predicted transposases and pseudogenes (Fig. 1; Table S8). Using Operon-mapper (61), five gene subclusters were predicted within the “Magic” gene cluster, each encoding for two to three proteins (Fig. 4A). Automated annotations from the NCBI and IMG provided few insights into their potential functions (Table 2) with one exception: the tenth consecutive gene was predicted to encode for 6-hydroxycyclohex-1-ene-1-carbonyl-CoA dehydrogenase (BamQ, Protein #11), a component of the ATP-independent benzoyl-CoA degradation pathway (Fig. 2). The detection of a neighboring transcription-terminating Rho factor in Experiment #3b (Protein #31) suggests that expression of the “Magic” gene cluster is tightly regulated.

Two other highly detected proteins (#32 and #33, ranked 18th and 4th in Experiment #3b, respectively) mapped to a second ORM2a genomic island of unknown function, designated the “Nanopod” gene cluster (Table 2; Table S8; Fig. 1 to 3), which includes four other expressed genes. Nanopods are tubular structures (<100 nm in diameter) that may facilitate nutrient transport across cell membranes or export toxicants (62, 63). Shetty et al. (62) found that phenanthrene induced nanopod formation in *Delftia acidovorans* Cs1-4 but not structurally similar hydrocarbons (naphthalene and toluene), and that deletion of its DUF1329 gene (DelCs14_1722) impaired growth on phenanthrene. BLASTP alignments suggest that Protein #33 is distantly related to the translated DelCs14_1722 gene sequence (21% amino acid identity), but the high expression of this ORM2a gene cluster and its possible relation to nanopod formation is intriguing.

### Comparative analyses of the “Magic” and “Nanopod” gene clusters to other genomes and proteomes

To determine whether other organisms harbor syntenic regions to the “Magic” and “Nanopod” gene clusters, translated protein sequences were searched against all reference genomes in NCBI and IMG databases (as of August 2024) using BLASTP. Four syntenic contigs were identified (Fig. 3; Table 3), along with six shorter contigs (comprising 2–3 genes) containing homologous genes with 51%–76% amino acid identity to those in the “Magic” gene cluster (Table S14). These genes also appear to be grouped into subclusters identical to those in the ORM2a genome (Fig. 3 and 4A). Nine of the ten contigs identified were affiliated with *Deltaproteobacteria* spp. from hydrothermal vent systems (64, 65). Hydrocarbons are important energy sources for microbial communities in these ecosystems and are produced primarily by geological processes (66). Hydrothermal vent MAG *Deltaproteobacteria* AUK045 is notable for containing syntenic contigs to both the “Magic” and “Nanopod” gene clusters (Fig. 3; Table 3). Additionally, genes encoding downstream benzoyl-CoA degradation and β-oxidation pathway components were detected on other contigs within AUK045 and two draft *Deltaproteobacteria* bins (Table S14), suggesting metabolic potential for aromatic compound degradation (64, 65).

**TABLE 3:** BLASTP search results for ORM2a “Magic” and “Nanopod” proteins against MAG4_ORM2b and publicly available sequence databases in GenBank and JGI (as of August, 2024)^*a*^

Protein no.	ORM2a	ORM2b_MAG4	*Pelotomaculum* candidate BPL MAG	Hydrothermal vent MAG AUK045	Hydrothermal vent bin B94_G9	Hydrothermal vent bin B7_G9	Hydrothermal vent MAG, Pescadero Basin	Wastewater MAG, Germany	Freshwater sediment MAG, Oklahoma
	This study	This study	Ga0073689 (IMG)	GCA_021163815.1	GCA_003646755.1	GCA_003646785.1	GCA_021160085.1	GCA_017996625.1	GCA_016935335.1
*Highly expressed "Magic" gene cluster putatively involved in benzene activation*									
23	**100%**	**96%**	**76%**	**72%**	**71%**	**71%**	25%	24%	27%
24	**100%**	**95%**	**76%**	**73%**		**73%**			
25	**100%**	**97%**	**74%**	**76%**				22%	
26	**100%**	**97%**	**70%**	**69%**	**66%**	28%	26%	29%	29%
27	**100%**	**94%**	**66%**	**62%**	**61%**			24%	
28	**100%**	**98%**	**74%**	**65%**	**72%**	**69%**			28%
29	**100%**	**98%**	**69%**	**60%**	**62%**	**62%**			29%
30	**100%**	**90%**		**61%**	51%				
31	**100%**	**90%**		**60%**	52%				
11	**100%**	**94%**	**64%**	27%		57%	21%	**62%**	
*Highly expressed "Nanopod" gene cluster putatively involved in benzene transportation across cell membrane*									
32	**100%**	**84%**		44%		26%	30%	23%	29%
33	**100%**	**94%**		**60%**	32%	36%	31%	23%	33%
34	**100%**	**82%**	32%	43%	31%	29%		28%	31%
35	**100%**	**92%**							
36	**100%**	**88%**		46%			31%	30%	30%
37	**100%**	**92%**		51%	33%		35%	36%	34%

^*a*^ NCBI accession numbers and percent amino acid identities are shown for the best BLASTP hits to select MAGs and bins with significant alignments (e-value < 1.0E–05, coverage ≥ 50%) to ORM2a proteins. Protein accession numbers and best BLASTP hit search results for other ORM2a proteins of interest and other reference genomes are provided in Table S14. Reference proteins that are highly similar to "Magic" and "Nanopod" ORM2a proteins (≥ 60% amino acid identity) are highlighted in bold.

*Pelotomaculum* candidate BPL (IMG GOLD accession number Ga0073689), the dominant benzene degrader in a sulfate-reducing enrichment culture from Poland (9, 35), provides another compelling comparison. Dong et al. (35) reconstructed its MAG in 2017 (2,986,7007 bp across 97 contigs, ~99% completeness) and published concurrent proteomic data from an active benzene-degrading subculture. A highly expressed *Pelotomaculum* contig (Ga0073689_145) produced eight significant alignments to the “Magic” gene cluster, exhibiting 64%–76% amino acid identity and e-values ≤ 1.0E-178 (Fig. 3; Table 3). The Ga0073689_145 contig also encodes a predicted benzoyl-CoA reductase subunit (BamB) and an enoyl-CoA hydratase, drawing parallels to the “Magic” gene neighborhoods in the ORM2a, ORM2b, and *Deltaproteobacteria* AUK045 MAGs (Fig. 3). These genomic and proteomic parallels are striking and suggest a conserved role for the “Magic” cluster in anaerobic benzene degradation.

### Predicted functions of the “Magic” proteins

Multiple sequence- and structure-based protein prediction tools were used to investigate the potential biochemical roles of proteins encoded by the “Magic” and “Nanopod” gene clusters. Search results for the “Magic” proteins are summarized below, in Fig. 4B and in Tables S14 to S16. Structure predictions for select “Magic” proteins and putative enzyme complexes are shown in Fig. 5. Phylogenetic trees were also constructed for each protein; however, most were too distantly related to characterized enzymes to yield meaningful functional insights.

“Magic” Gene Subcluster #1 encodes Proteins #22 and #23 (Fig. 4A). All prediction tools converged on the annotation of Protein #22 as a novel AMP-forming acyl-CoA synthetase (Fig. 4B). Phylogenetic analysis placed Protein #22 and its homologs from other anaerobic benzene degraders in a distinct subfamily of acyl-CoA synthetases, separate from well-characterized enzymes such as benzoyl-CoA ligase and acetyl-CoA synthetase (Fig. S7). DeepFRI GO predictions (67) further suggested that Protein #22 can bind to organic cyclic compounds such as benzene (Table S16b). Since Proteins #22 and #43 are predicted to use similar cofactors (e.g., ATP/AMP, CoA), these reactions may be coupled, with ATP generated by one reaction driving the other (Fig. 2). Protein #23 contains a canonical “hot dog” domain typical of (*R*)-specific enoyl-CoA hydratases (68) and is likewise predicted to bind cyclic organic molecules (Tables S15 and S16b). Enoyl-CoA hydratases catalyze the second step of β-oxidation (69), converting trans-2-enoyl-CoA molecules into 3-hydroxyacyl-CoA. Structure predictions for Proteins #22 and #23 are shown in Fig. S8A and B, respectively.

Gene Subcluster #3 encodes Proteins #27 and #28, predicted to form a class III CoA-transferase complex (Fig. 4B; Fig. S8C). These enzymes operate via a ping-pong mechanism to transfer CoA groups between carboxylic acids. They are known to act on diverse substrates, including oxalate and benzylsuccinate, which is the fumarate addition product of toluene (70). Proteins #29 and #30, encoded on Gene Subcluster #4, were poorly annotated by most tools but may form a separate enzyme complex (Fig. 4B; Fig. S8D). Interestingly, MotifFinder (71) detected weak homology in both proteins to domains in PaaZ, a bifunctional ring-hydrolyzing enzyme involved in phenylacetyl-CoA degradation (72) (Table S15). Gene Subcluster #5 encodes BamQ (Fig. 4B) and two proteins—a GNAT family N-acetyltransferase and a transposase-associated protein—that were not detected in the proteome (Fig. 3). Homologs of these two proteins are absent in the MAGs of *Pelotomaculum* candidate BPL or *Deltaproteobacteria* AUK045, suggesting that they may not participate in benzene degradation.

Proteins #24–26, encoded on Gene Subcluster #2, were the three most highly detected proteins in the OR proteomes (Fig. 4A; Table 2). AlphaFold 3 predictions (73) suggest that these proteins form a heterotrimeric complex most structurally similar to glycolate dehydrogenase (GlcDEF) from *E. coli* among biochemically characterized enzymes curated in Swiss-Prot (Fig. 5). GlcDEF is a non-bifurcating enzyme complex composed of two flavoproteins (GlcDE) and an Fe-S protein (GlcF) that catalyzes the oxidation of glycolate to glyoxylate via hydride transfer to a flavin cofactor, with electrons subsequently transferred to a quinone pool (74). Consistent with this structural relationship, multiple prediction tools classified Proteins #24 and #26 as flavoproteins with potential activity toward small organic substrates (Fig. 4B). DeepFRI (67) predicted that Protein #24 can bind to cyclic organic compounds and FAD (Table S16b), the latter of which is supported by AlphaFold 3 models (Fig. 5A). A putative substrate-binding pocket adjacent to the FAD-binding site was also identified in Protein #24 (Fig. S9). Although structural modeling and alignment of the Protein #24–26 and GlcDEF complexes show conservation of the overall complex architecture (Fig. S10), corresponding subunits share ≤23% amino acid identity. Therefore, it remains unclear whether similar substrates are recognized by both complexes.

Phylogenetic analyses placed Protein #25 within a clade of heterodisulfide reductase (Hdr)-like proteins distantly related to any characterized enzyme (Fig. S11 and S12). Members of this protein family are typically involved in electron transfer rather than direct substrate catalysis (74, 75). GO term predictions from PROST (76) suggest that Protein #25 may have 2-hydroxyacid dehydrogenase activity (Table S16a). Protein #25 contains four cysteine-rich motifs consistent with Fe-S cluster binding (Fig. 5A), but two of these motifs are missing conserved cysteine residues that may impact or differ the electron transfer properties from those of canonical Hdr subunits (Fig. S13).

Overall, functional inferences suggest that the “Magic” gene cluster encodes a suite of previously uncharacterized enzymes predicted to interact with cyclic organic substrates. The abundant detection of corresponding proteins during methanogenic benzene degradation, together with their conserved synteny among obligate anaerobic benzene degraders, strongly implicates the gene cluster in anaerobic benzene degradation and potentially its elusive activation mechanism.

### Predicted function of the “Nanopod” proteins

Protein prediction tools suggest that the “Nanopod” gene cluster encodes a transmembrane complex (Fig. 2; Tables S15 and S16). Protein #32 most closely resembles a porin, involved in passive substrate transport, whereas Protein #37 (a predicted RND superfamily efflux protein) appears to be involved in active transport. GO term searches in PROST (76) and DeepFRI (67) suggest that Protein #37 and possibly Protein #34 (an uncharacterized cytoplasmic protein) can bind to organic cyclic structures (Table S16a and b). Most tools agreed that Protein #35 is a universal stress factor. Membrane stress can result from partitioning of hydrophobic compounds such as benzene into lipid bilayers (77). Collectively, it is plausible that the “Nanopod” gene cluster facilitates benzene movement across cell membranes, although the direction(s) of movement cannot yet be confirmed (Fig. 2).

### Phylogenomic placement of the ORM2a and ORM2b_MAG4 genomes

**Fig 6:**
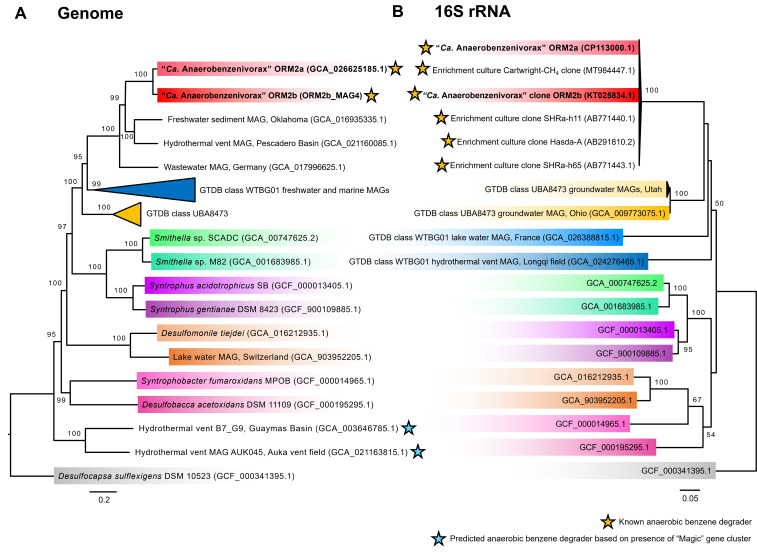
Taxonomic classification of ORM2a and reference *Desulfobacterota* species based on (**A**) genome and (**B**) 16S rRNA gene maximum likelihood phylogenetic trees. Sequences from this study are highlighted in bold. *Desulfocapsa sulflexigens* was used to root both trees. Bootstrap values <60% are not shown.

The taxonomic classification of ORM2a and ORM2b has been revised multiple times in response to ongoing updates in microbial taxonomy (4, 40, 41). New phylogenomic analyses (Fig. 6A) placed the MAGs within an uncharacterized clade of *Desulfobacterota*, distinct from previously assigned taxonomic groups, including the candidate order Sva0485 and GTDB classes WTBG01 and UBA8473, as well as a clade including *Deltaproteobacteria* MAG AUK045. The ORM2a and ORM2b clade, which appears to represent a novel class within the *Desulfobacterota*, also includes three environmental MAGs, one of which (GCA_021160085.1) was recovered from yet another hydrothermal vent system (64). While these MAGs harbor some downstream functional potential to degrade benzoyl-CoA and its intermediates (Table S14), they lack key genes implicated in benzene activation (Table 3).

A complementary 16S rRNA gene-based phylogenetic tree (Fig. 6B) revealed a consistent clustering pattern, clearly indicating that ORM2a and ORM2b form a monophyletic group of benzene-degrading specialists (4). These organisms, enriched from geographically diverse origins, share ≥ 99% 16S rRNA gene sequence identity and have only been observed to degrade benzene under sulfate-reducing and/or methanogenic conditions (37, 78, 79). Unfortunately, full genomes are lacking for most of these organisms. Taken together, the phylogenomic evidence presented here strongly supports the delineation of ORM2a and ORM2b as members of a novel genus within the *Desulfobacterota*. We propose the name “*Candidatus* Anaerobenzenivorax” (An.ae.ro.ben.ze.ni.i.vor’ax. Gr. pref. *an*, not or without; Gr. n. *aer*, air; N.L. neut. n. *benzenum*, benzene, L. adj. *vorax,* devouring, N.L. neut. n. Anaerobenzenivorax, benzene-devouring without air. The proposed taxonomy is currently being registered in SeqCode (80).

## DISCUSSION

Many efforts have been made to elucidate the mechanisms of anaerobic benzene activation. Recent advances in metagenomics, proteomics, and *in silico* protein structure prediction are now enabling deeper insights into these processes. Because benzene is the only known growth-supporting substrate for organisms such as “*Ca*. Anaerobenzenivorax” strains ORM2a and ORM2b, conventional differential growth experiments are not feasible, and we must instead infer these mechanisms from meta-omics data sets. As shown in this study, consistent detection of a highly expressed, conserved gene cluster across multiple years and independent laboratories strongly implicates the “Magic” gene cluster in initiating benzene metabolism via a novel mechanism leading to benzoyl-CoA formation. However, the available data cannot yet define the underlying reaction chemistry, electron acceptor(s), or intermediates. Below, we outline a working hypothesis of the reaction mechanism (Fig. S14) purely intended to guide future biochemical testing.

### Proposed mechanism of anaerobic benzene activation catalyzed by ORM2a

The high activation energy required to functionalize benzene, particularly in the absence of oxygen, remains a central challenge. Direct single-electron transfer and reductive activation of benzene seems implausible because the reduction potential required to form a benzene radical anion is estimated to be 1 V more negative than that of benzoyl-CoA (81), placing it well outside the biological redox window. Oxidative activation of benzene by direct hydrogen-atom abstraction is similarly unlikely, as the C–H bond in benzene (bond dissociation energy ≈113 kcal/mol) is even stronger than the C–H bond in methane (≈105 kcal/mol) (82). Several radical-generating systems could, in principle, meet this challenge, including SAM-dependent, glycyl radical-, and flavin-based chemistries. Our proteomic and structural predictions indicate that Proteins #24–26 form a novel oxidoreductase complex with FAD and Fe–S-associated features, making flavin-mediated redox chemistry a plausible component of the activation process.

Although flavin radicals are generally less reactive toward direct C–H bond abstraction than glycyl- or thiyl-based radicals, flavin-dependent enzymes can nevertheless catalyze chemically demanding transformations by stabilizing high-energy intermediates and avoiding direct homolytic cleavage of aromatic C–H bonds (83, 84). For example, the 4-hydroxybutyryl-CoA dehydratase from *Clostridium aminobutyricum* is proposed to generate an enzyme-bound flavin semiquinone radical (FADH•) upon substrate binding via an internal one-electron transfer; this FADH• is then suggested to initiate the thermodynamically unfavorable dehydration of 4-hydroxybutyryl-CoA through an allylic ketyl radical intermediate (83). Flavoenzymes can also catalyze reactions with no net redox changes (85), a property that could be advantageous in strictly anaerobic, energy-limited metabolisms where internal electron recycling is favored.

We envision that benzene activation in the OR consortium may involve a radical-mediated coupling reaction between benzene and an unidentified co-substrate (Fig. S14). Based on functional predictions (Tables S4b, S15, and S16) and structural models (Fig. S8 and S9), the FAD-containing Protein #24 could represent the catalytic subunit initiating benzene activation. In contrast, Hdr-like Protein #25 appears better suited for intracomplex electron transfer and potentially electron cycling. The electron source required to generate the flavin semiquinone intermediate is currently unknown but could derive from benzene during a coupled redox interaction, or from transient oxidation of an associated co-substrate. Although highly speculative, we believe that glutaconyl-CoA should be explored as a candidate co-substrate based on the reproducible and comparatively high abundance of proteins associated with the conversion of benzoyl-CoA to glutaconyl-CoA (Proteins #1–17) but not for downstream β-oxidation steps (Proteins #18–21) (Fig. 2). The overall reaction between benzene and glutaconyl-CoA could yield acetyl-CoA and cinnamate, with the latter subsequently oxidized to benzoyl-CoA (Fig. S14). Electron balance calculations (Table S17) show that the proposed activation reaction is redox-neutral, with electrons transiently redistributed within the enzyme complex, most likely via flavin and Fe–S cofactors, and ultimately returned to the activation products.

### Transformation of the proposed benzene activation intermediate to benzoyl-CoA

If cinnamate is the product of the initial benzene activation reaction, the most plausible next steps include coenzyme A activation followed by modified β-oxidation (86), yielding benzoyl-CoA and releasing an additional mole of acetyl-CoA (Fig. S14). Stoichiometry indicates that three moles of acetyl-CoA and three moles of H_2_ or equivalent are produced per mole of benzene oxidized (87). To balance this stoichiometry, Proteins #22 and #27–28 may function in appending CoA moieties to cinnamate and a downstream intermediate, enabling β-oxidation to proceed. The third mole of acetyl-CoA is expected to be released by a downstream thiolase (Protein #14) during the conversion of 3-hydroxypimelyl-CoA to glutaryl-CoA (Fig. 2; Fig. S14). Electrons are balanced in this proposed pathway (Table S17).

β-oxidation consists of four consecutive steps: dehydrogenation, hydration, oxidation, and thiolysis. Multiple protein prediction tools implicate Proteins #23 and #29–30 in hydration reactions (Fig. 4B; Table S13 and S14). However, the “Magic” gene cluster lacks clear candidates for dehydrogenation, oxidation, and thiolysis steps. It remains unclear whether these functions are carried out by enzymes encoded elsewhere in the genome or by uncharacterized proteins within the “Magic” cluster that catalyze analogous reactions.

### Closing remarks

There is little doubt that the “Magic” gene clusters in ORM2, *Pelotomaculum* candidate BPL, and specific hydrothermal vent organisms participate in anaerobic benzene degradation, and likely in its elusive, energetically difficult activation step. By contrast, the “Nanopod” gene cluster, present in ORM2a, ORM2b_MAG4, and *Deltaproteobacteria* AUK045, may facilitate benzene transport across the cell membrane. Given that the MAGs of most anaerobic benzene degraders surveyed were incomplete, it is possible that homologous “Nanopod” genes were missed. It is telling that the only genomes with both the “Magic” and “Nanopod” clusters belonged to strict anaerobes with apparent specialization in benzene metabolism. The location of both clusters on genomic islands (Fig. 1) suggests acquisition via horizontal gene transfer. Now that these unique gene clusters have been identified, we hope the broader research community will help confirm their functions and continue the search for homologous systems in other data sets and enrichment cultures.

## MATERIALS AND METHODS

Detailed descriptions of all materials, experimental procedures, and computational analyses are provided as supplemental material.

## SUPPLEMENTAL MATERIAL

Supplemental material aem.02083-25-s0001.pdf Supplemental methods, Table S1, and Fig. S1 to S14.

Supplemental tables aem.02083-25-s0002.xlsx Tables S2 to S17.

ASM does not own the copyrights to Supplemental Material that may be linked to, or accessed through, an article. The authors have granted ASM a non-exclusive, world-wide license to publish the Supplemental Material files. Please contact the corresponding author directly for reuse.
