# Supplementary material for: Identification of a highly expressed gene cluster likely coding for benzene activation enzymes in a methanogenic enrichment culture
Source: Appl Environ Microbiol. 2026 Mar 27;92(4):e02083-25. doi: 10.1128/aem.02083-25 (PMC13101534; doi:10.1128/aem.02083-25)
Supplement: Supplemental material — Supplemental methods, Table S1, and Fig. S1 to S14. [file aem.02083-25-s0001.pdf]

Supporting Information (SI)

Identification of a Highly Expressed Gene Cluster Likely Coding for Benzene Activation Enzymes in a Methanogenic Enrichment Culture

Courtney R. A. Toth[1], Olivia Molenda[1], Camilla Nesbø[1], Fei Luo[1‡], Cheryl E. Devine[1], Xu Chen[1], Kan Wu[1], Johnny Z. Xiao[1], Rishika Puri[1], Shen Guo[1], Nancy Bawa[1], Po-Hsiang Wang[1,2], Yifeng Wei[2], Robert Flick[1], and Elizabeth A. Edwards[1*]

[1]Department of Chemical Engineering and Applied Chemistry, University of Toronto, Toronto, Ontario, M5S 3E5, Canada.

[2]Signapore Institute of Food and Biotechnology Innovation, Agency for Science, Technology and Research (A*STAR), Singapore, 138669, Singapore

[‡]Current Address: Liven Proteins Corporation, Mississauga, Ontario, L5L 1C6, Canada

[*]For correspondence, e-mail: elizabeth.edwards@utoronto.ca; Tel. (+1) 416 946 3506; Fax (+1) 416 978 8605.

Number of Pages: 33

Materials and Methods

Number of Supplementary Texts: 1

Number of SI Tables: 16

Number of SI Figures: 14

Number of Supporting Files: 3

TABLE OF CONTENTS

Materials and Methods……………………………………………………………………..5

Supplementary Text….…..…………………………………………………………………..11

Text S1. Supplementary features of the ORM2a genome…………………………………….…11

Supplementary Tables (Tables S2-S17 are provided in an accompanying Excel file)……………12

Table S1. History of the methanogenic benzene-degrading OR consortium…………………………12

Table S2. Relative abundance of archaea, bacteria, and ORM2 in OR maintenance cultures sampled between 2019-2024

Table S3. Summary of OR metagenomes and metagenome-assembled genomes (MAGs)

Table S4. General features of the ORM2a MAG

Table S5. Complete and incomplete KEGG metabolic pathways identified in the ORM2a genome. The table is separated into a summary (**Table S5a**) and more detailed information (**Table S5b**) into the KEGG pathways identified

Table S6. Putative transporter genes identified in the ORM2a MAG

Table S7. Operons in the ORM2a MAG putatively associated with syntrophic metabolic processes and hydrogen evolution

Table S8. Putative genomic islands (GI) and prophage regions identified in the ORM2a MAG

Table S9. Putative transposase genes and pseudogenes identified in the ORM2a MAG

Table S10. Putative insertion sequences (IS) identified in the ORM2a MAG

Table S11. Proteins identified using LC-MS/MS sequencing of OR crude lysates and SDS-PAGE gel slices

Table S12. Computational and statistical analysis of liquid chromatography tandem mass spectrometry (LC-MS/MS) output. The table is separated by peptide (**Table S12a**) and protein (**Table S12b**) information

Table S13. Functional genes identified in the OR metagenomes putatively associated with the metabolism of benzoyl-CoA and related aromatic structures

Table S14. BLASTP search results for ORM2a proteins of interest against MAG4_ORM2b and publicly available genomes in GenBank and JGI

Table S15. Best homology search results for ORM2a proteins coded by "Magic" and "Nanopod" gene clusters

Table S16. Best Gene Orthology (GO) term search results for ORM2a proteins coded by "Magic" and "Nanopod" gene clusters. The table is separated by sequence-based (**Table S16a**) and structure-based (**Table S16b**) search results

Table S17. Electron balances for anaerobic benzene degradation pathways catalyzed by ORM2a

Supplementary Figures……………………………………………………………………16

Figure S1. Subculturing history of the methanogenic OR consortium's "OR-b" lineage…..……16

Figure S2. Benzene degradation, methane production, and microbial community dynamics in a representative methanogenic OR consortium subculture……………………………………...17

Figure S3. Output results from Ori-Finder 2022……………………………………………...18

Figure S4. The average nucleotide identity between the closed genome of ORM2a and a draft MAG putatively belonging to ORM2b………………………………………………………...19

Figure S5. Polyacrylamide gel electrophoresis of proteins extracted from Experiment #3b……20

Figure S6. Maximum likelihood consensus trees showing the affiliation of predicted OR consortium *bam* gene products to reference protein sequences from select anaerobic aromatic degraders……………………………………………………………………………………21

Figure S7. Maximum likelihood consensus trees showing the affiliation of Proteins #22, #43 and homologs to known and predicted AMP-binding acyl-CoA synthetase enzymes……..………..22

Figure S8. AlphaFold 3 predicted models of Protein #22, Protein #23, the Protein #27-28 complex, and the Protein #29-30 complex……………………………………………………….……23

Figure S9. Supplementary AlphaFold 3 models of the Protein #24-26 enzyme complex………...24

Figure S10. Structural model of the Protein #24-26 enzyme complex superimposed with the glycolate dehydrogenase complex GlcDEF in *Escherichia coli*………………………………25

Figure S11. Maximum likelihood consensus trees showing the affiliation of Protein #25 and homologs to known and putative heterodisulfide reductase enzymes…………………………....26

Figure S12. Maximum likelihood consensus trees of known and putative heterodisulfide reductase enzymes……………………………………………………………………………………..27

Figure S13. Multiple sequence alignment of cysteine-rich motifs in Protein #25 and selected iron-sulfur homologs from methanogens and *Escherichia coli*……………………………………….28

Figure S14. Conceptual model of anaerobic benzene degradation by ORM2a…………………30

References……………………………………………………………………………….31

Supporting Files (available in figshare: https://doi.org/10.6084/m9.figshare.27312285.v2)

File S1. FASTA file of OR proteomics database

File S2. AlphaFold 2 structural models of ORM2a "Magic" and "Nanopod" proteins

File S3. AlphaFold 3 structural models of ORM2a "Magic" enzyme complexes

MATERIALS AND METHODS

Culture maintenance

The OR consortium was enriched from samples from an oil refinery in Oklahoma in 1995 (1) and has been maintained in a near-identical manner ever since. Briefly, cultures are grown in a defined pre-reduced anaerobic mineral medium (2, 3) and are fed benzene (~5-40 mg/L) once every 4-6 weeks. The OR consortium has been subcultured many times, resulting in the emergence of five distinct culture lineages (4, 5). The "OR-b" lineage, illustrated in Figure S3, is the most well characterized with a doubling time about 30 days (3-10) and was surveyed in this study. A large scale (>100 liters) bioaugmentation culture known as DGG-B (3, 9) was derived from the OR-b lineage. Though the OR consortium has been amended with other substrates including benzoate and toluene, only benzene has been shown to support the growth of ORM2a and ORM2b (6, 11).

Sequencing, assembly, and annotation of OR metagenomes and MAGs

Between 2010-2017, the metagenomes of three OR-b subcultures designated OR-b1A, DGG1A, and DGG-0 were sequenced. The OR-b1A (2010) metagenome was sequenced using Illumina paired-end technology (2×100 bp) and was partially assembled in ABySS v.1.3.2 (12) with contigs binned using varying kmer lengths. Details are available in Chapter 5 of Devine's PhD thesis (6). Metagenomic sequencing and assembly of DGG1A (2016) and DGG-0 (2017) was described as part of a Microbiology Resource Announcement by Toth et al. (8). Briefly, hybrid assembly of short-read (Illumina paired-end) and long-read (PacBio) shotgun sequences was utilized to produce long, high quality contigs. Following binning, three draft MAGs including ORM2a were refined into complete circularized MAGs (cMAGs) (8). The correct assembly of each cMAG genome was verified by read mapping, and the average sequence depth for the ORM2a genome was 1,384× (8, 13).

Taxonomy was assigned to all draft and complete MAGs using release 214 of the Genome Taxonomy Database (GTDB) and GTDB tool kit (v.2.3.2) using the classify_wf workflow (8, 14, 15). In this study, the ORM2a genome (CP113000.1) and a medium-quality MAG putatively belonging to ORM2b, (ORM2b_MAG4, 3,214,903 bp across 378 contigs) were reclassified using release 220 of GTDB and GTDB-Tk v.2.4.0 (discussed later). Seventy-one medium-to-high quality MAGs including ORM2b_MAG4 were retained. All metagenomic assemblies and complete MAGs were submitted to NCBI and/or IMG for automated gene calling and functional annotation. Incomplete (noncircular) MAGs were deposited to figshare (13) and in this study were annotated using RASTtk (16) in March, 2022. Data availability including accession numbers and all functional annotation pipelines used is provided in Table S3.

Analysis of the ORM2a genome

To assess the metabolic potential of the ORM2a genome, coding sequences predicted by the NCBI Prokaryotic Genome Annotation Pipeline were uploaded to BlastKOALA for automatic KEGG Orthology (KO) assignment (17), then organized into metabolic pathways using the Reconstruct tool within KEGG Mapper (18, 19). Next, the mummer2circos package in GitHub (https://github.com/metagenlab/mummer2circos) (20) was used to generate a circular plot of the ORM2a cMAG and to compare its homology with ORM2b_MAG4 (Figure 1). Two-way average nucleotide identity (ANI) analysis of both genomes was performed using an online calculator (http://enve-omics.ce.gatech.edu/ani/). Ori-Finder 2022 (21) was used to identify the genomes' origin of replication (*oriC*). IslandViewer 4 (22) and PHASTER (23) were used to identify genomic islands and prophage sequences, respectively. Functional annotations and hidden Markov model assignments (from NCBI and IMG) were used to identify transposase genes. Putative

insertion sequences were identified using ISfinder (24). Operon-mapper (25) was used to identify operons in the ORM2a genome.

Protein extraction and LC-MS/MS analysis

Between 2010-2018, three proteomics experiments were performed on three OR-b lineage subcultures (OR-b1C, OR-b, and OR-b1A; see Figure S3). Culture information, including culture volumes extracted and benzene degradation rates at the time of sampling, are provided in Table 1. To avoid oxygen contamination, all containers used were incubated in an anaerobic chamber (supplied with a gas mix of 10% H_2, 10% CO_2 and 80% N_2) for >24 hours prior to use, and all steps were performed at 4 °C unless otherwise specified.

Protein extractions for Experiment #1 (OR-b1C, 2010) and Experiment #2 (OR-b, 2011) were conducted by Devine (6). Cell pellets from 150-200 mL of culture were harvested by centrifugation (8,000 x g, 20 min), flash-frozen using liquid nitrogen, and stored at -80°C until further processing. On the day of protein extraction, thawed cells were resuspended in a lysis buffer solution (100 mM Tris-HCl [pH 8.0], 5% w/v glycerol, 10 mM EDTA, 1 mM PMSF [phenylmethylsulfonyl fluoride], and 5 mM dithiothreitol [DTT]) and sonicated at 23-30W for 10 min in pulse mode (1 s ON/1 s OFF for 1 min, 1 min OFF, repeat). Proteins in the crude lysate were recovered using phenol [pH 8.0] and precipitated with 100 mM ammonium acetate in methanol. After centrifuging (10,000 x g, 20 min) and discarding the supernatant, the protein pellets were washed with acetone, resuspended in a 6 M urea solution (pH 8.0), then subjected to an 18-24 hr in-solution digestion with porcine trypsin (19). The digestion was stopped using a solution of 2% trifluoroacetic acid (TFA) and 20% acetonitrile. The resulting peptides were purified using PepClean^{TM} Spin Columns (Pierce Biotechnology) according to the manufacturer's procedure.

In Experiment #3 (OR-b1A, 2018), cell pellets from two 50 mL volumes of culture (10,000 x g, 15 min) were suspended in an anoxic sodium dodecyl sulfate (SDS)-containing lysis buffer (50 mM Tris-HCl [pH 7.6], 2% w/v SDS, and 50 mM DTT) and sonicated at 23-30W for 10 min in pulse mode (1 s ON/1 s OFF for 1 min, 1 min OFF, repeat). Proteins from one crude lysate (Experiment #3a) were processed by filter-aided sample preparation (FASP) using Microcon-10 kDa centrifugal filter units (Millipore) as outlined by Wiśniewski et al. (26), followed by an 18 hr on-filter digestion with porcine trypsin at 37 °C. The second crude lysate (Experiment #3b) was mixed with 2× SDS gel-loading buffer (50 mM Tris-HCl [pH 6.8], 10% SDS [sodium dodecyl sulfate], 40% glycerol, 3 mM bromophenol blue, and 500 mM DTT]) and separated on a 15% SDS-PAGE gel run for 45 min at 200 V. Visualization of the resulting gel revealed a long protein smear with no distinct bands (Figure S2), which was sliced into five equal sections then destained and digested with trypsin as described in Shevchenko et al. (27).

LC-MS/MS analysis of Experiments #1 and #2 peptides (6) was performed at the SickKids SPARC BioCentre using an LTQ Orbitrap mass spectrometer (Thermo Fisher Scientific). Run parameters are summarized in Tang et al. (28). LC-MS/MS analysis of Experiment #3 peptides was conducted at the University of Toronto BioZone Mass Spectrometry Facility. Briefly, 5 µL liquid samples of peptides were separated on a 15cm PicoTip Emitter (New Objective) packed with ReproSil-Pur C18-AQ 3 µm resin (Dr. Maisch GmbH). The flow rate was set to 250 nL/min and the eluents used were (A) water containing 0.1% formic acid, and (B) acetonitrile containing 0.1% formic acid. The gradient started at 0% B, followed by a linear gradient to 10% B over 5 min, a linear gradient to 40% B over 88 min, a linear gradient to 95% B over 2 min, a hold at 95% B for 10 min, a linear gradient to 0% B over 1 min, and a final hold of 0% B for 14 min (total runtime of 120 min). MS detection was conducted using a Q-Exactive Orbitrap mass spectrometer

(Thermo Fisher Scientific) equipped with a nanoelectrospray ionization probe operating in positive ionization mode, with a spray voltage of 2.8 kV, capillary temperature of 275°C, and S-lens radio frequency level of 55. Full MS data was acquired with an m/z range of 400-2,000, mass resolution of 70,000, automatic gain control (AGC) target of 1.0E+06, and a maximum injection time of 30 milliseconds. MS2 was gathered using a data dependant TOP10 approach with a mass resolution of 17,500, AGC target of 5.0E+04, maximum injection time of 50 ms.

Protein identification

Raw mass spectrometry data from all three experiments were converted into mzXML using MSconvert (29) then uploaded into Version 2020.11.12.1 of X! Tandem (The GPM, thegpm.org) for peptide and protein discovery. Spectra were screened against a large library of protein sequences (1,595,050 entries) with a mass tolerance of <0.4 Da for the fragment ions and <20 ppm for the precursor ion. The library, provided in File S1 and overviewed in Table S3, contained 1,106,523 translated gene sequences called by IMG (three metagenomic assemblies and 3 cMAGs), NCBI (3 cMAGs), and RAST-tk (67 draft MAGs). The database also included 488,411 reverse-translated (decoy) protein sequences from the DGG0 metagenome to calculate a false discovery rate, and 116 entries from common human/laboratory contaminants. Scaffold v. 4.4.1.1 (Proteome Software Inc.) was used to visualize and validate MS/MS based peptide and protein identifications. Peptide and protein identifications were accepted if they could be established at greater than 95% and 99% probability, respectively (30, 31). Proteins sharing peptide evidence were grouped into clusters.

Functional and structural prediction of ORM2a proteins detected in high abundances

Protein identity was verified using protein BLAST searches against all other OR metagenomic assemblies and MAGs, as well as reference (meta)genomic sequences in NCBI (nr), JGI (IMG/M),

and UniProt (UniProt KB reference proteomes + Swiss-Prot). Next, the sequence-based tool MotifFinder (32) was used to identify conserved domains in each protein. Tertiary protein structures were predicted using the AlphaFold2.ipynb tool within Colab (33, 34), then compared to homologous structures and protein domains within the Dali server's Protein Data Bank (35) and DeepFRI (36). All AlphaFold 2 protein structural models generated are provided in File S2. Additionally, AlphaFold 3 (37) was used to predict enzyme complex structures for select proteins of interest (File S3). Maximum likelihood trees were constructed in RAxML v.8.2.1.1 within Geneious v.8.1.9 (38, 39) to corroborate predicted protein functions where possible. Protein sequences were also sent to the laboratory of Dr. Christine Orengo (University College London) for independent functional analysis using CATH v.4.4 (40) and PROST (41).

Phylogenomic analyses

Phylogenomic analysis of the closed ORM2a genome and ORM2b_MAG4 was performed in v.1.8.8 of GToTree (42). Briefly, we retrieved all representative genomes from c__UBA8473 and c__WTBG01 from GTDB v.220, as well as closely related genomes from selected *Desulfobacterota*, then extracted target genes using the Proteobacteria.hmm single copy gene set (119 genes) within GToTree. *In silico* translated protein sequences were aligned using MUSCLE (43) and concatenated. A maximum likelihood tree was constructed from the concatenated alignment using IQ-TREE v.2.3.6 (44) with the LG+F+I+R5 substitution model, inferred as best model by IQ-TREE, and 100 bootstraps. For comparative purposes, a second maximum likelihood tree was constructed using 16S rRNA gene sequences extracted from each representative GTDB genome (if available) and from clone sequences of known/predicted anaerobic benzene degraders. *Desulfocapsa sulflexigens* DSM 10523 (GCA_000341395.1) was included as an outgroup.

Text S1. Supplementary features of the ORM2a genome

Twelve possible genomic islands were identified in the ORM2a cMAG, of which two are likely prophage sequences and 9 contain putative transposase genes (Figure 1 and Table S9). An additional 47 transposase genes and 63 insertion sequences-like structures were scattered across the cMAG (Figure 1 and Tables S9-S10), hinting that the ORM2a genome has been shaped by numerous rearrangement events and may explain the absence of a definitive terminus region. A similarly high transposase content was found in the cMAG of "*Ca*. Nealsonbacteria" DGGOD1a, a predicted necromass recycler (5). No CRISPR elements, virulence factors, or antimicrobial resistance genes were identified. Genes coding for type IV pili and a Che-type chemotaxis system may be involved in bacterial motility.

Only 29 complete anabolic pathways were identified in the ORM2a cMAG, including biosynthesis pathways for 7 amino acids and 4 vitamins (Table S5a). In contrast, 197 genes with putative transport functions were identified (Table S6) – including transporters for vitamin B_{12} and undefined amino acids – hinting that ORM2a imports most of its nutrients from extracellular sources, perhaps from polymeric substances or from direct exchanges with other microorganisms as theorized in recent microscopy and genomic investigations of the OR culture (5, 10). Similar transporter gene counts were recovered from the genome of *Syntrophus acidotrophicus* SB, a fermentative benzoate-degrading anaerobe whose central and peripheral metabolic pathways are also largely incomplete (45, 46).

Table S1. History of the methanogenic benzene-degrading OR consortium. Findings most relevant to this study are bolded.

Reference and year	Major milestones and discoveries	Significance for understanding the OR consortium
Nales et al., 1998 (1)	• Observed anaerobic benzene degradation linked to various electron-accepting conditions in microcosms from multiple sites.	• Established microcosms from an Oil Refinery (OR) site in Oklahoma, USA, which led to the enrichment of the OR consortium
Ulrich and Edwards, 2003 (2); Ulrich, PhD Thesis (3)	• Described nine anaerobic benzene-degrading enrichment cultures from 4 locations.<br>• Constructed 16S rRNA clone libraries.<br>• Demonstrated anaerobic benzene degradation stoichiometrically coupled to nitrate reduction, sulfate reduction and methanogenesis.<br>• Reported growth yield estimates and doubling times.	• Provided the first formal definition and characterization of the OR consortium.<br>• **Established Deltaproteobacterium ORM2 (*Oil Refinery Methanogenic Culture Clone #2*) as a key organism of interest.**<br>• **Reported a 30-day doubling time for the OR culture.**
Mancini et al., 2003 (47)	• Reported carbon and hydrogen isotopic fractionation for the OR consortium compared to other anaerobic benzene-degrading cultures.	• Represented the first use of compound-specific isotope analysis (CSIA) to probe anaerobic benzene activation mechanisms.<br>• Provided indirect evidence that anaerobic benzene activation differs fundamentally from aerobic mechanisms.
Ulrich et al, 2005 (48)	• Detected metabolites during $^{13}C_6$-benzene degradation in the methanogenic OR consortium and in a nitrate-reducing benzene-degrading culture.<br>• Evaluated the ability of the OR consortium to degrade toluene and benzoate; no appreciable degradation was observed.	• Based on detection of ^{13}C-phenol, ^{13}C-benzoate, and ^{13}C-toluene, hydroxylation and methylation were initially proposed as possible benzene activation mechanisms – a hypothesis later shown to be incorrect as results could not be reproduced.<br>• Demonstrated that the OR consortium is highly specialized for benzene metabolism
Mancini et al., 2008 (7)	• Used dual-parameter (carbon and hydrogen) isotope fractionation plots to compare benzene activation mechanisms in various anaerobic benzene-degrading enrichments.<br>• **Methanogenic and sulfate-reducing cultures exhibited consistently higher dual-isotope slopes than nitrate-reducing cultures.**	• **Provided compelling evidence for existence of at least two anaerobic benzene activation mechanisms.**<br>• Demonstrated that the OR consortium and other methanogenic/sulfate-reducing cultures likely use a benzene activation mechanism distinct from that of nitrate-reducing cultures.

Reference and year	Major milestones and discoveries	Significance for understanding the OR consortium
Devine, PhD Thesis (6)	• Generated the first metagenome assembly of the methanogenic OR consortium.<br>• **Discovered at least two distinct strains of ORM2** within the consortium, along with a hydrogen-utilizing *Methanoregula* sp. and a previously unidentified organism classified within Candidate Division OD1.<br>• Produced the first proteomics datasets for the OR culture and **demonstrated that ORM2 expresses a class II benzoyl-CoA reductase (Bam) pathway during benzene degradation, consistent with canonical anaerobic aromatic degradation pathways**.	• Represented a critical step in identifying ORM2a and ORM2b as prime candidate benzene degraders and linking specific proteins to benzene metabolism.<br>• **Established the first functional genomic and proteomic framework for studying benzene degradation in the OR consortium.**<br>• Findings were not initially published outside of a thesis due to uncertainty regarding the initial benzene activation step and low protein yields.
Luo et al., 2016 (4)	• Applied 16S rRNA gene amplicon sequencing and qPCR for the first time to characterize the methanogenic OR consortium.<br>• Identified ORM2a and ORM2b as the two most abundant bacteria, consistently detected across all surveyed subcultures.<br>• **Detected a highly abundant bacterium of previously unknown function, OD1 ("*Ca*. Nealsonbacteria"), along with two methanogens (*Methanothrix* and *Methanoregula*) in every subculture.**<br>• **Time-series growth experiments and yield calculations confirmed that ORM2a and ORM2b are the only bacteria whose growth directly coincides with benzene degradation.**	• **Provided unequivocal evidence that ORM2a and ORM2b are the primary benzene-metabolizing organisms in the OR consortium.**<br>• Revealed substantial microbial "dark matter" in the OR consortium, including OD1/"*Ca.* Nealsonbacteria", establishing high-priority targets for future ecological and functional investigation.<br>• Provided the first phylogenetic evidence that methanogenic benzene degraders from globally distributed ecosystems are closely related.
Luo, PhD Thesis (11)	• Evaluated numerous cultivation strategies and medium formulations in attempts to increase anaerobic benzene degradation rates; only marginal improvements were observed, and none were adopted for long-term culture maintenance.<br>• Successfully switched a longstanding nitrate-reducing benzene-degrading culture to methanogenic conditions, resulting in the enrichment of an ORM2-like bacterium.<br>• **Performed preliminary enzyme assays on the methanogenic OR consortium; notably, no benzoate–CoA ligase activity was detected.**	• Established key methodological considerations for culturing ORM2-containing consortia and provided the foundation for future scale-up of the methanogenic OR culture to large volumes.<br>• Reinforced the central ecological role of ORM2 and ORM2-like organisms in methanogenic benzene degradation.<br>• Enzyme assay results suggested that benzoate is not an intermediate during in the OR consortium and that **benzene is likely not activated via carboxylation, distinguishing it from nitrate-reducing benzene degraders.**

Reference and year	Major milestones and discoveries	Significance for understanding the OR consortium
Toth et al., 2021 (9)	• A subculture of the methanogenic OR consortium was successfully scaled up to volumes >100 L, generating a stable large-scale lineage designated DGG-B.<br>• In microcosms containing benzene-contaminated sediments, bioaugmentation with DGG-B reliably accelerated anaerobic benzene biodegradation.<br>• Demonstrated a direct correlation between ORM2 abundance and methanogenic benzene degradation rates.<br>• Showed that nitrate-reducing benzene degradation is associated with growth of a distinct bacterial clade, (Thermincolales).	• Scaling the culture to large volumes enabled high-biomass sampling and facilitating future studies. **Scaled up lineage of the OR consortium renamed DGG-B in honour of anaerobic hydrocarbon degradation pioneer Dr. Dunja Grbić-Galić.**<br>• Promising results from bioaugmentation trials with DGG-B at field sites across North America strengthen links between abundances of ORM2/ORM2-like bacteria and rates of methanogenic benzene degradation in real-world environments.<br>• Clear differentiation of methanogenic and nitrate-reducing benzene degraders reinforced the existence of distinct benzene activation strategies in nature.
Guo et al., 2022 (3)	• Laboratory exposure of DGG-B to oxygen caused rapid decay of ORM2 and enrichment of an aerobic benzene-degrading *Pseudomonas* species.<br>• After oxygen was depleted, the consortium required ~1 year for ORM2 populations to recover sufficiently to reestablish methanogenic benzene degradation.	• **Confirmed that the anaerobic benzene biotransformation mechanism used by ORM2 is independent of oxygen** and fundamentally distinct from established aerobic degradation pathways.
Toth et al., 2023 (8)	• Metagenomes from two DGG-B subcultures were sequenced using long-read (PacBio) and short-read (Illumina) platforms.<br>• Hybrid assembly and iterative bin refinement enabled complete genome closure of ORM2a, OD1/“Ca. Nealsonbacteria”, and a *Methanoregula* species. The closed ORM2a genome exhibited extremely high mean contig coverage (992.7 ± 112.4×).<br>• Reconstructed 71 additional metagenome-assembled genomes (MAGs), including a draft ORM2b MAG.	• **The closed ORM2a genome and draft ORM2b MAG provide blueprints for deciphering the mechanism of anaerobic benzene activation.**<br>• The diverse set of reconstructed MAGs enhanced understanding of the metabolic roles and ecological interactions of other members of the OR consortium, including microbial “dark matter” such as “*Ca*. Nealsonbacteria”.

Reference and year	Major milestones and discoveries	Significance for understanding the OR consortium
Chen et al., 2023 (5); Chen, PhD Thesis (15)	• Supplied the methanogenic OR consortium with various electron donors to identify potential growth substrates for "*Ca*. Nealsonbacteria."<br>• Observed a ~10-fold increase in "Ca. Nealsonbacteria" cell numbers when the culture was amended with crude cell lysate.<br>• Fluorescence in situ hybridization (FISH) and cryo-transmission electron microscopy (cryo-TEM) revealed that "Ca. Nealsonbacteria" cells were physically attached to larger *Methanothrix* cells.	• Demonstrated that "Ca. Nealsonbacteria" likely exhibits an epibiont (episymbiotic) lifestyle with acetoclastic methanogens (*Methanothrix)*, consistent with metabolic predictions from its closed genome.<br>• This interaction may play a key role in biomass recycling within the OR consortium.
Chen et al., 2025 (10); Chen, PhD Thesis (15)	• Used time-series FISH to visualize the spatial organization of ORM2 cells under varying benzene concentrations.<br>• At benzene concentrations <60 mg/L, ORM2 cells formed tight aggregates with other bacteria and methanogenic archaea.<br>• At high benzene concentrations (90–120 mg/L), cell aggregation decreased markedly, with ORM2 appearing more dispersed.	• Demonstrated that the methanogenic OR consortium may exhibit concentration-dependent spatial structuring, the functional significance of which is not yet known.

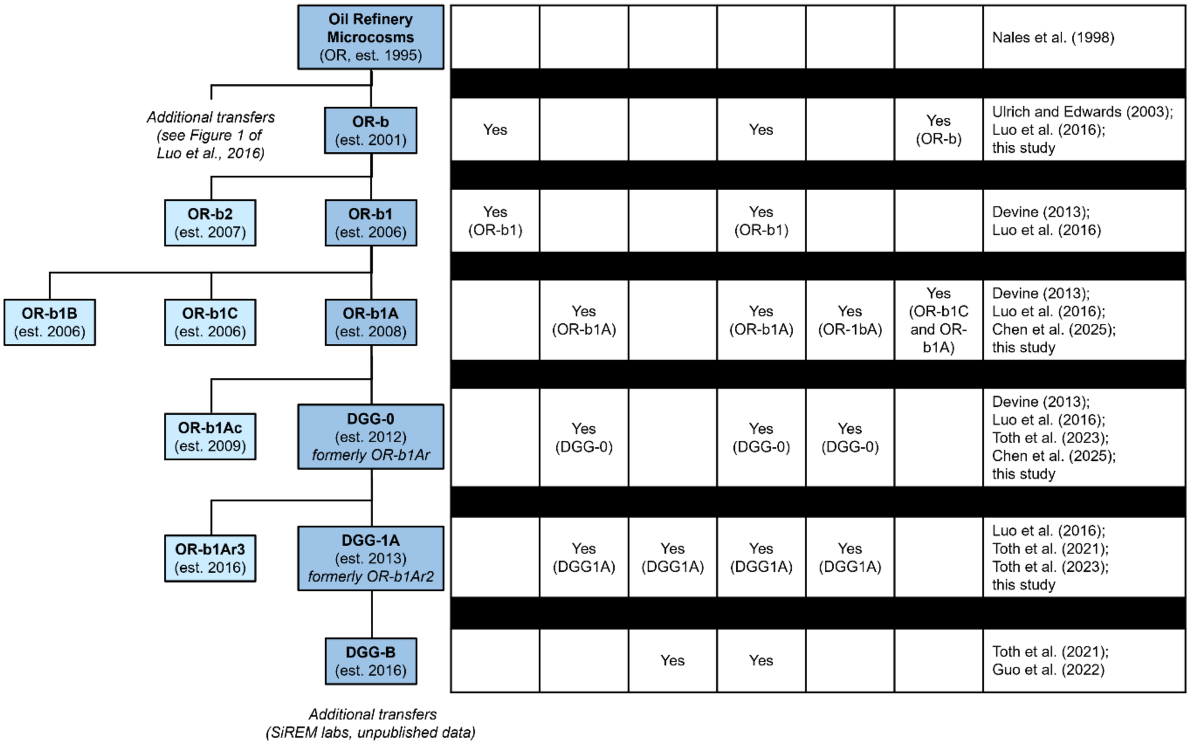

Culture	16S rRNA clone library	16S rRNA pyrosequencing	16S rRNA Illumina sequencing	16S rRNA gene-based qPCR	Metagenomics	Proteomics	Reference(s)
Oil Refinery Microcosms (OR, est. 1995)							Nales et al. (1998)
OR-b (est. 2001)	Yes			Yes		Yes (OR-b)	Ulrich and Edwards (2003); Luo et al. (2016); this study
OR-b1 (est. 2006)	Yes (OR-b1)			Yes (OR-b1)			Devine (2013); Luo et al. (2016)
OR-b1A (est. 2008)		Yes (OR-b1A)		Yes (OR-b1A)	Yes (OR-1bA)	Yes (OR-b1C and OR-b1A)	Devine (2013); Luo et al. (2016); Chen et al. (2025); this study
DGG-0 (est. 2012)		Yes (DGG-0)		Yes (DGG-0)	Yes (DGG-0)		Devine (2013); Luo et al. (2016); Toth et al. (2023); Chen et al. (2025); this study
DGG-1A (est. 2013)		Yes (DGG1A)	Yes (DGG1A)	Yes (DGG1A)	Yes (DGG1A)		Luo et al. (2016); Toth et al. (2021); Toth et al. (2023); this study
DGG-B (est. 2016)			Yes	Yes			Toth et al. (2021); Guo et al. (2022)

Figure S1. Subculturing history of the methanogenic OR consortium, including microbial and meta-omics analyses performed to date.

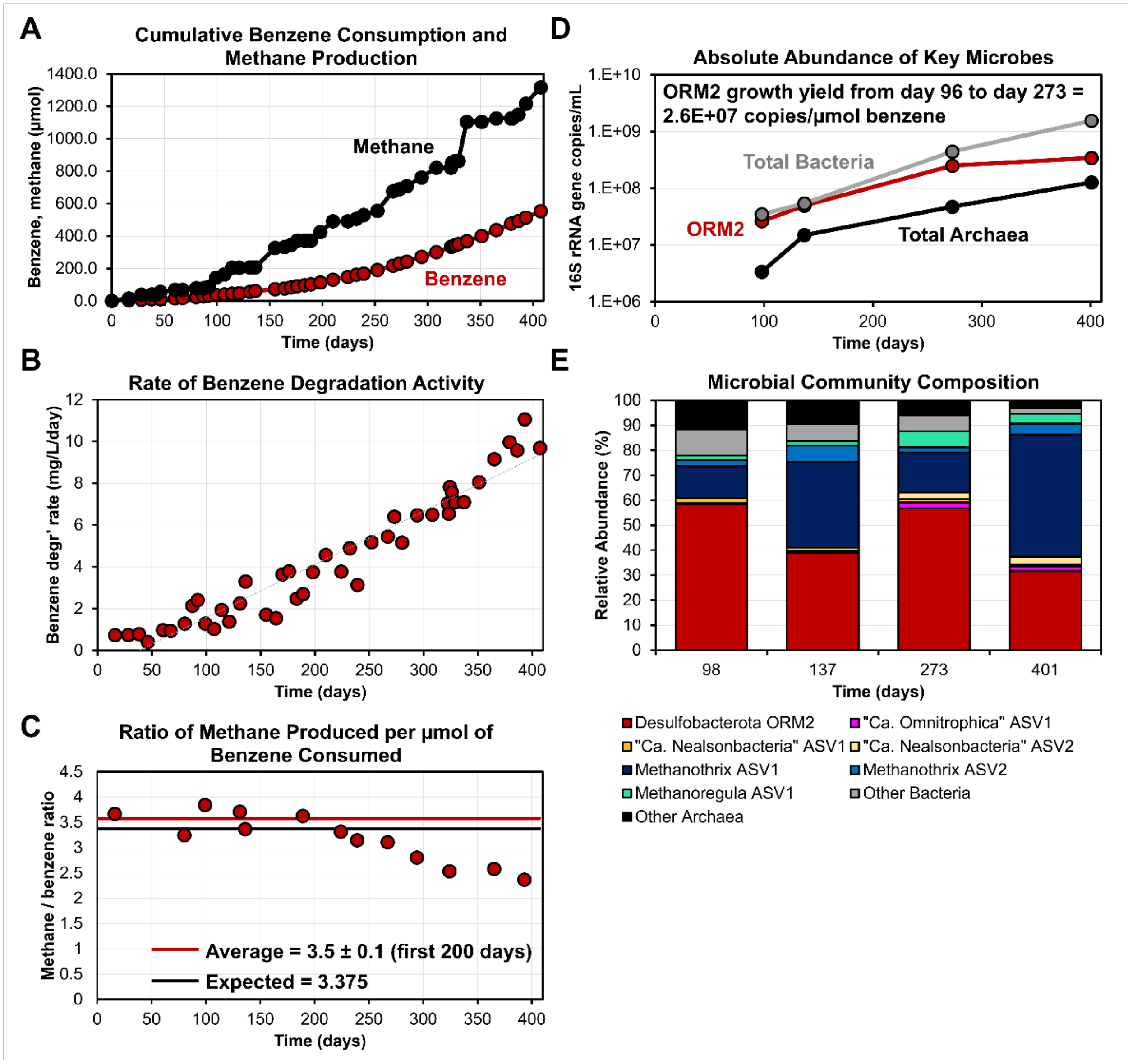

Figure S2. Benzene degradation, methane production, and microbial community dynamics in a representative methanogenic OR consortium subculture. Data are from a DGG-B subculture ("Bottle 4") previously described by Guo et al. (3). The culture was re-amended with 20-150 mg/L benzene every 2-3 weeks, any time benzene concentration decreased below 5 mg/L. Left panels (A, B) show temporal trends in benzene degradation activity and stoichiometric methane production. Right panels (D, E) show quantitative PCR and 16S rRNA gene amplicon sequencing trends for key members of the OR consortium. In panel (C), the methane-to-benzene ratio is shown to match predicted stoichiometry; this ratio decreased slightly after 200 days and was likely due to excess gas production and less accurate measurements.

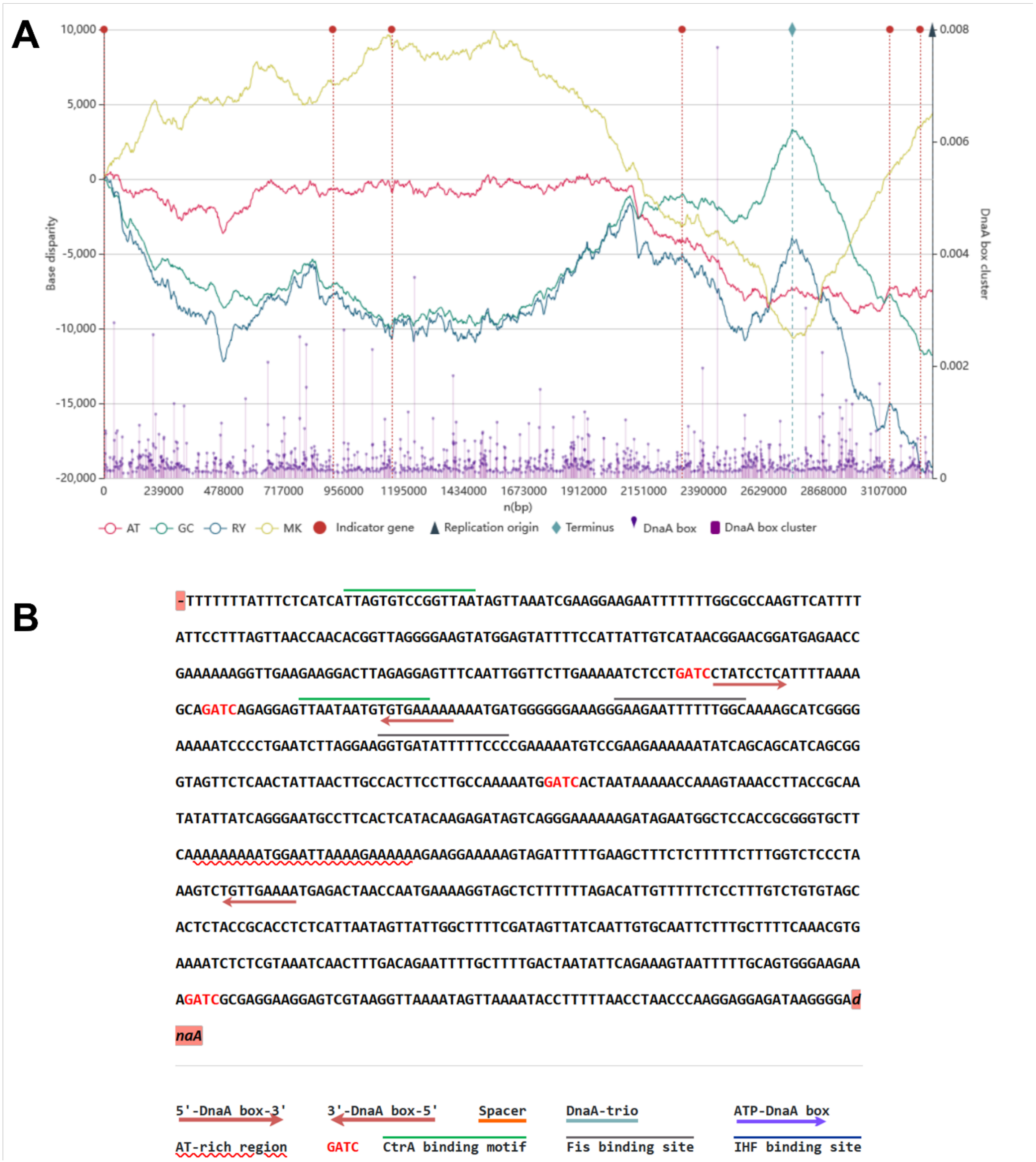

Figure S3. Output results from Ori-Finder 2022 (21). The top panel (A) shows RY, MK, AT and GC disparity curves of the ORM2a genome, the location of 5 indicator genes (*dnaA*, 790…2,136 nt; *hemE*, 911,117…912,208 nt; *dnaN*, 1,146,408…2,304,626 nt; *mnmG*, 3,131,767…3,133,644 nt, *hemB*, 3,251,271…3,252,257 nt), the predicted replication origin (*oriC*), the maximum of GC disparity (2,742,827 nt), DnaA boxes (i.e., DnaA binding sites), and DnaA box clusters. The bottom panel (B) shows the *oriC* sequence including specific binding motifs. The *dnaA* gene is located immediately downstream of *oriC*.

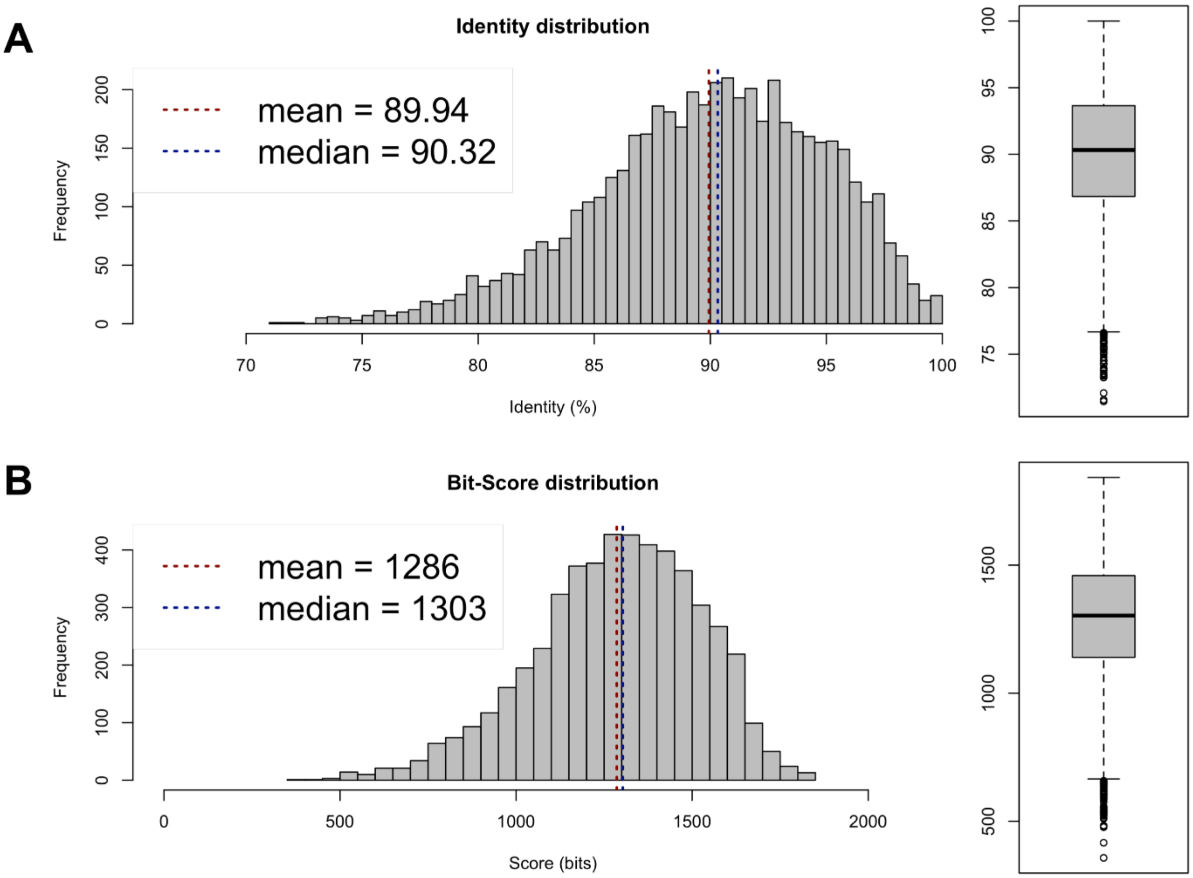

Figure S4. The average nucleotide identity (ANI) between the closed genome of ORM2a (CP113000.1) and a draft MAG putatively belonging to ORM2b (ORM2b_MAG4). Panel (A) shows the best reciprocal BLAST hits (two-way ANI) between each genome pair where aligned coding regions share ≥70% identity. Panel (B) shows the statistical significance (bit-score) of each alignment. Figures and ANI values were generated using the ANI calculator developed by the Kostas lab (http://enve-omics.ce.gatech.edu/ani/).

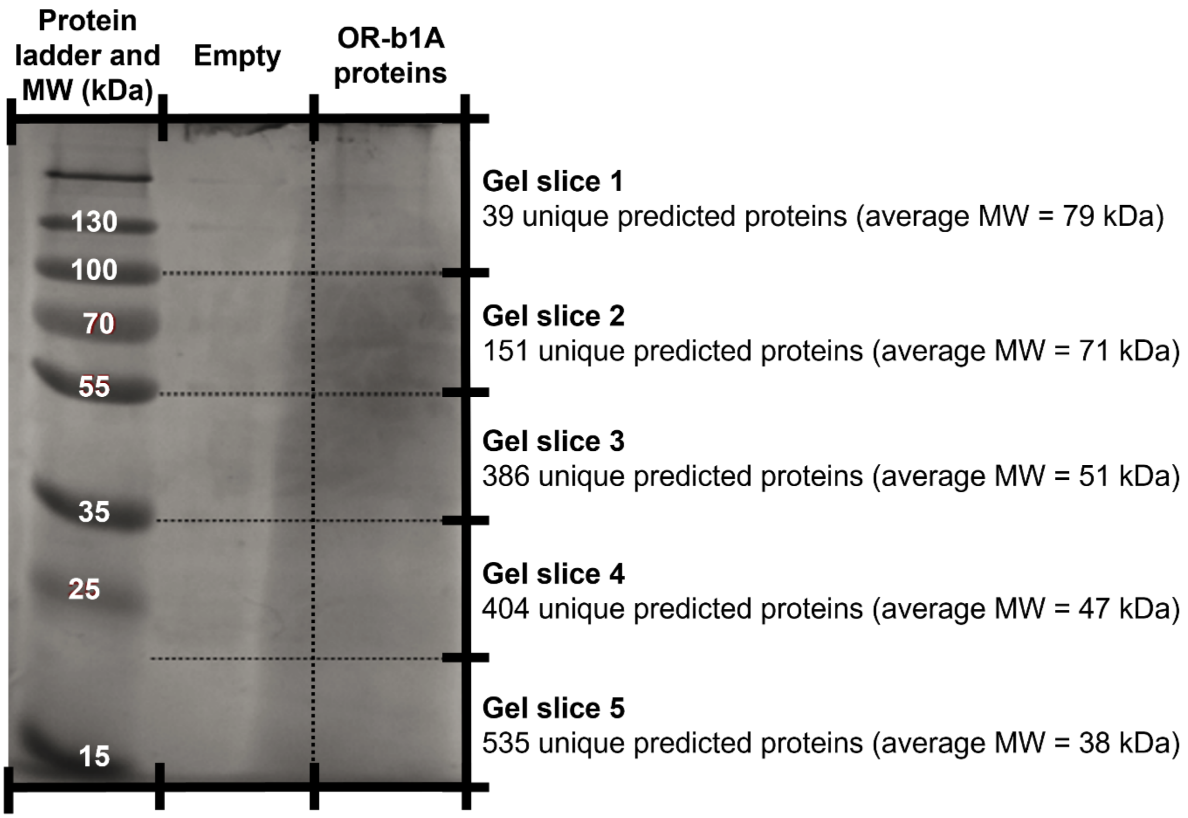

Figure S5. Polyacrylamide gel electrophoresis (SDS-PAGE) of proteins extracted from Experiment #3b. Proteins from a commercial ladder (left-most lane) and the crude extract from OR-b1A (right-most lane) are shown. Dotted lines show the approximate locations where the OR-b1A protein band was sliced in preparation for in-gel trypsin digestion and LC-MS/MS sequencing (Experiment #3b). MW = molecular weight.

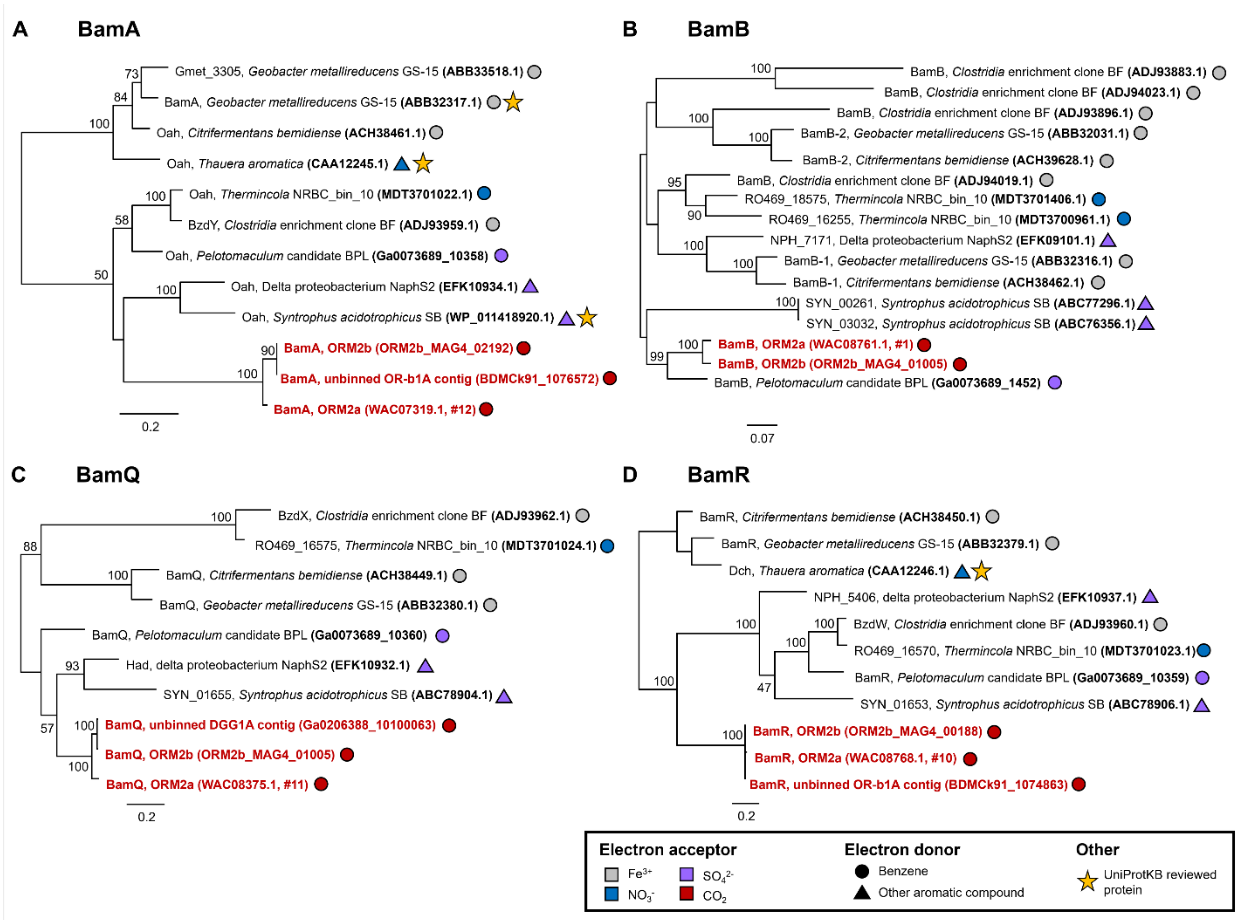

Figure S6. Maximum likelihood consensus trees showing the affiliation of predicted OR consortium *bam* gene products – (A) 6-oxo-cyclohex-1-ene-carbonyl-CoA hydrolase, (B) benzoyl-CoA reductase subunit b, (C) 6-hydroxycyclohex-1-ene-1-carbonyl-CoA dehydrogenase, and (D) cyclohexa-1,5-diene-1-carbonyl-CoA hydratase – to reference protein sequences from select anaerobic aromatic degraders. Sequences highlighted in red are from this study. Proteins marked with stars have some experimental evidence of protein function and have been reviewed by UniProtKB. Bootstrap values <50% are not shown. GenBank accession numbers are provided for most proteins; IMG accession numbers are provided for *Pelotomaculum* candidate BPL and the OR-b1A metagenome. Amino acid sequences for ORM2b_MAG4 are available in Table S14. Multiple sequence alignment statistics for each protein are also available in Table S14.

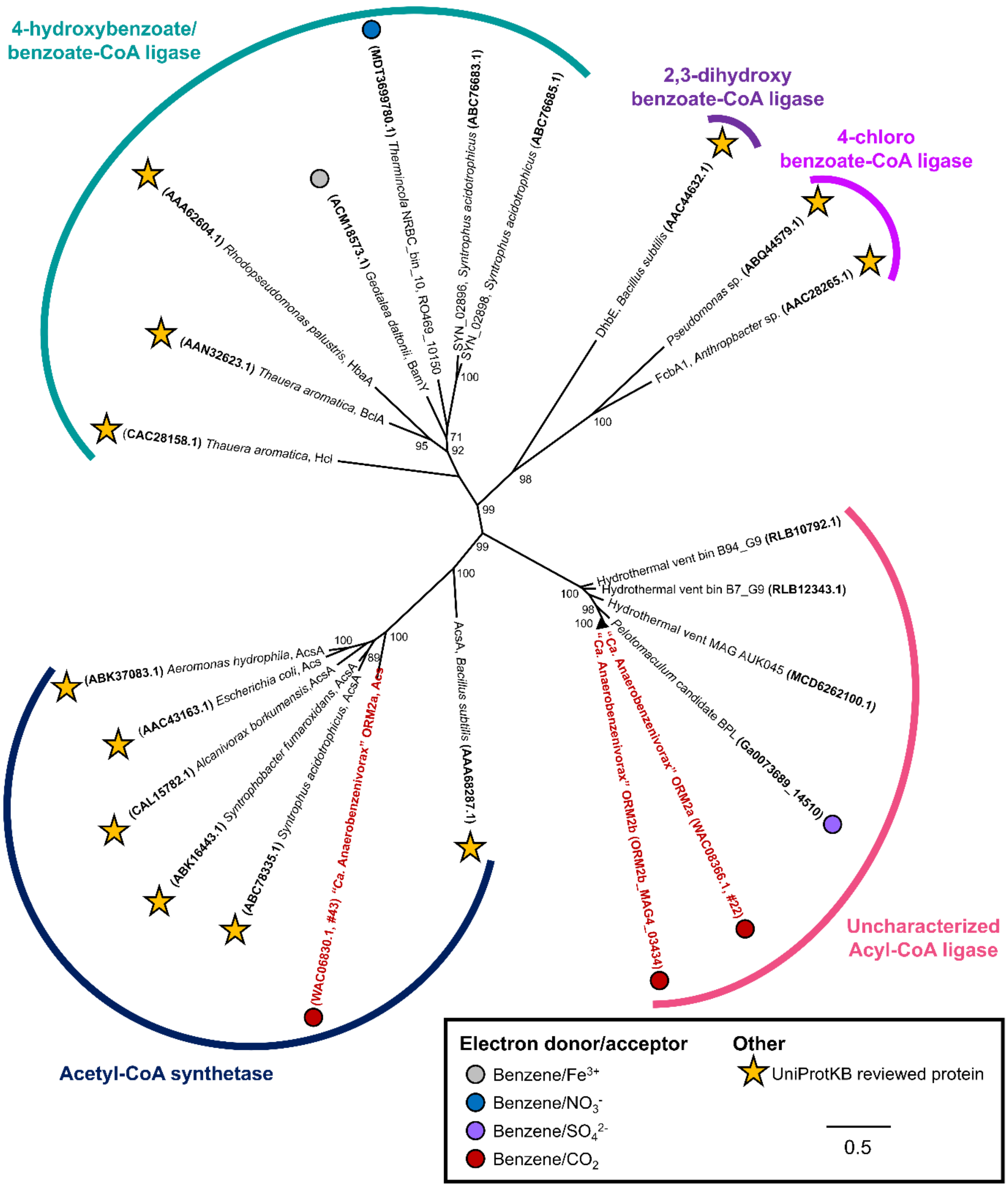

Figure S7. Maximum likelihood consensus trees showing the affiliation of Proteins #22, #43 and homologs to known and predicted AMP-binding acyl-CoA synthetase enzymes. Proteins marked with stars have some experimental evidence of protein function and have been reviewed by UniProtKB. Bootstrap values <70% are not shown. Multiple sequence alignment statistics for Proteins #22 and #43 are also available in Table S14.

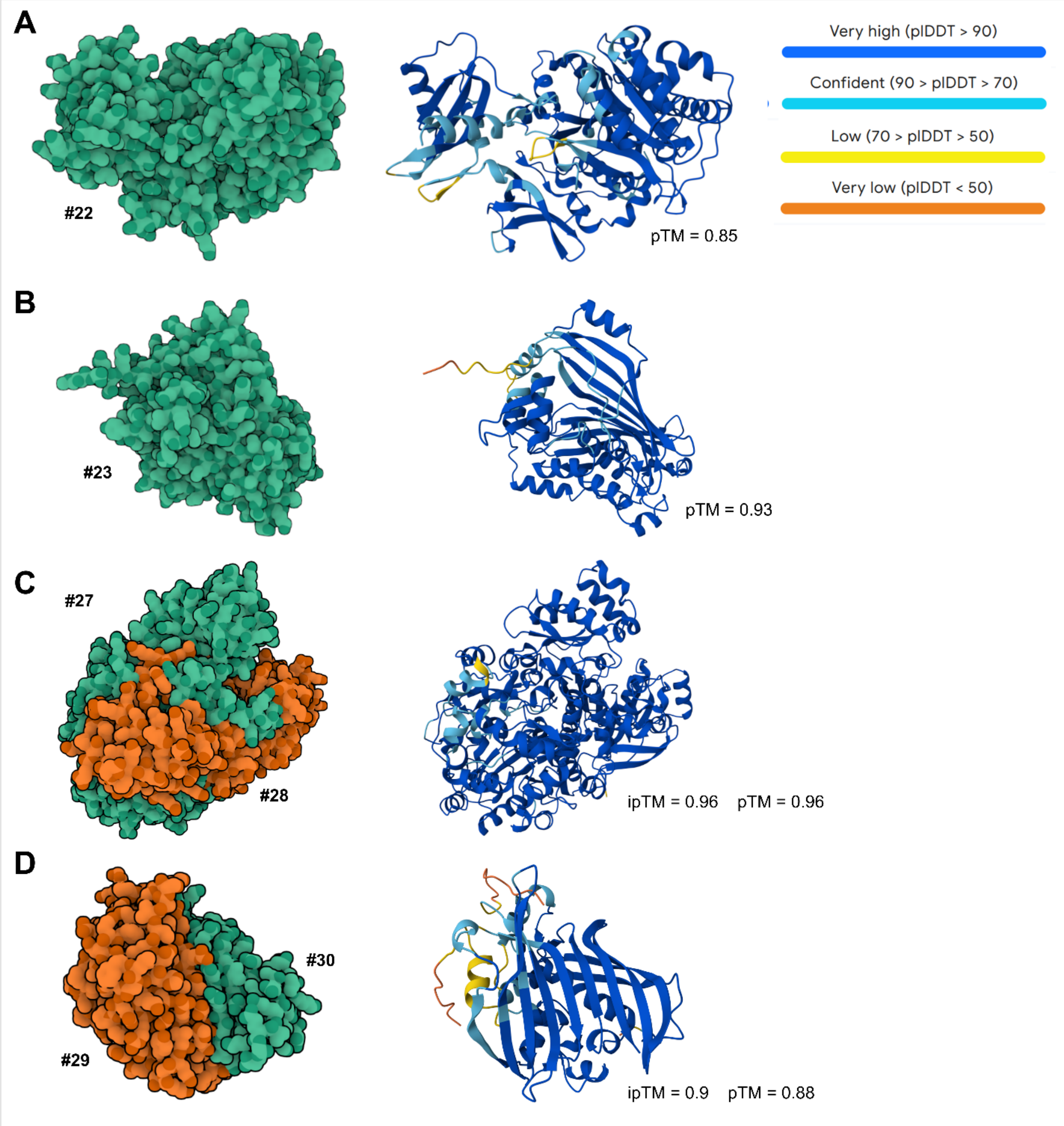

Figure S8. AlphaFold 3 predicted models of (A) Protein #22, (B) Protein #23, (C) the Protein #27-28 complex, and (D) the Protein #29-30 complex. Surface and ribbon models of each structure are shown. The colour outputs of each ribbon model depict predicted Local Distance Difference Test (pIDDT) confidence estimate of each atom on a 0-100 scale, where a higher value indicates higher confidence. The predicted template modeling (pTM) score and the interface predicted template modeling (ipTM), which estimates the folding accuracy of predicted structures and protein complexes, respectively, is also shown on a 0-1 scale, where a higher value indicates higher confidence.

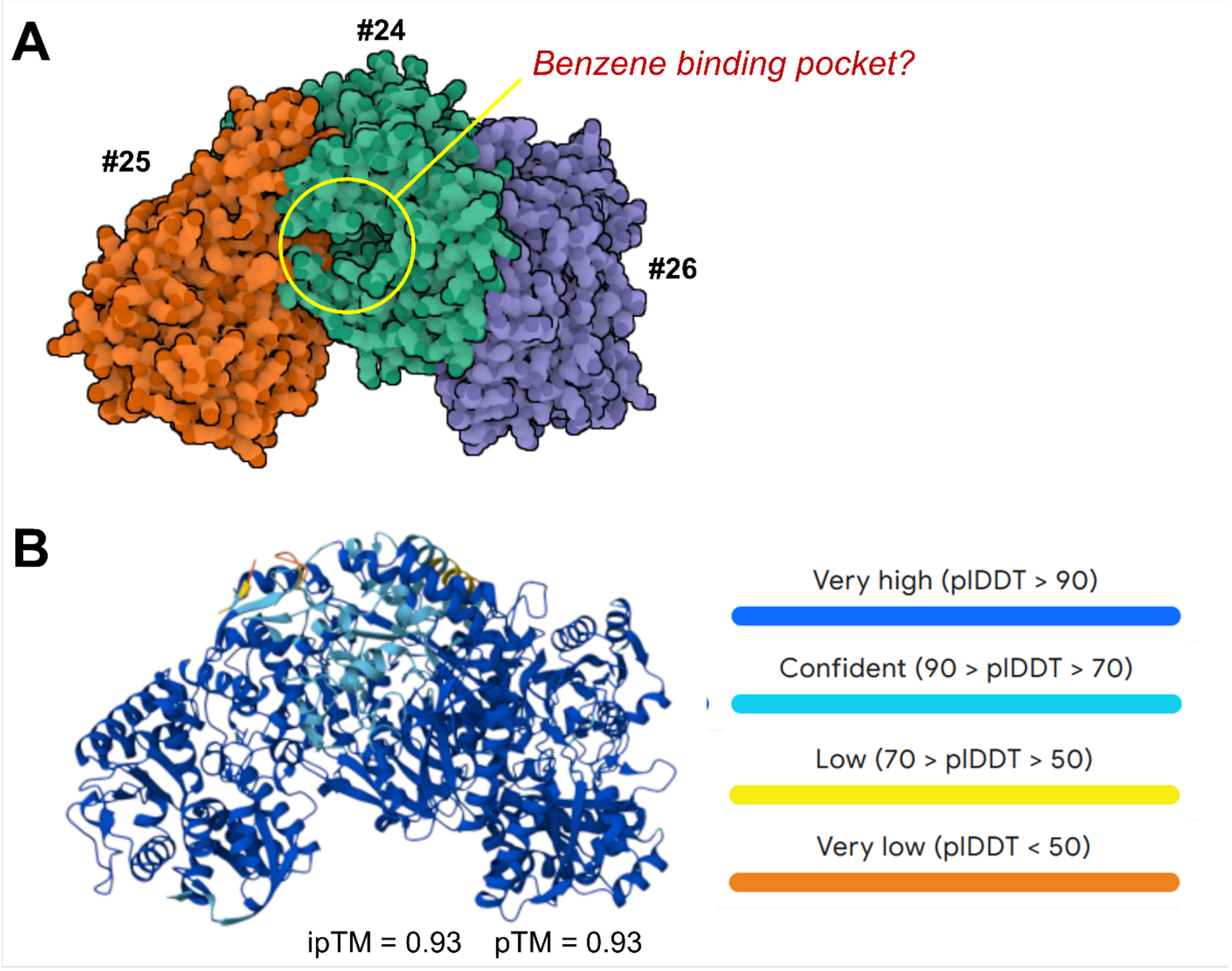

Figure S9. Supplementary AlphaFold 3 models of the Protein #24-26 enzyme complex. Surface (A) and ribbon (B) models of each structure are shown. A putative binding pocket is shown in the surface model of Protein #24. The colour outputs of the ribbon model depicts the predicted Local Distance Difference Test (pIDDT) confidence estimate of each atom on a 0-100 scale, where a higher value indicates higher confidence. The predicted template modeling (pTM) score and the interface predicted template modeling (ipTM), which estimates the folding accuracy of predicted structures and protein complexes, respectively, is also shown on a 0-1 scale, where a higher value indicates higher confidence.

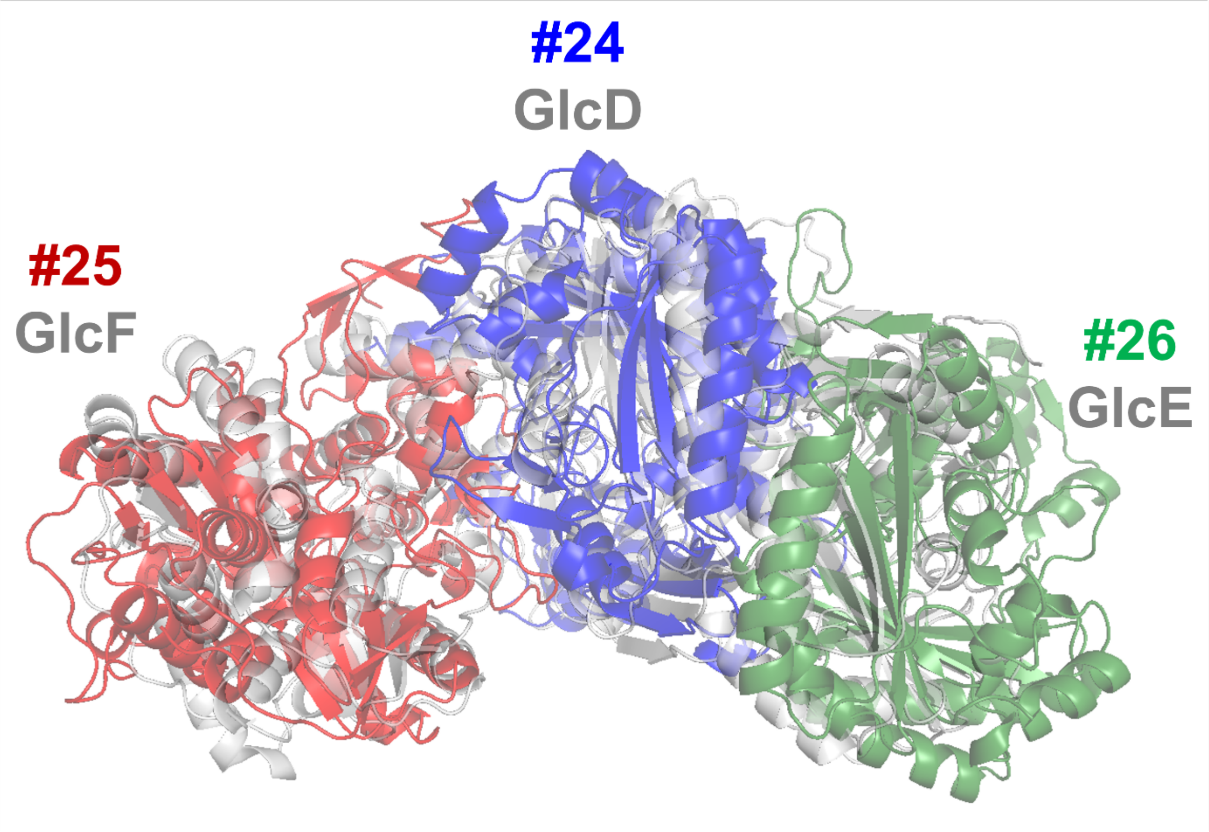

Figure S10. Structural model of the Protein #24-26 enzyme complex (colored) superimposed with the glycolate dehydrogenase complex GlcDEF in *Escherichia coli* (grey). Structures were superimposed using MM-align (49), yielding a template model (TM)-score of 0.71 (normalized over #24–26) and a root-mean-square deviation (RMSD) of 4.59 Å across 1,183 aligned residues. RMSD reflects the average distance between aligned backbone atoms and provides a measure of structural similarity; TM-scores are reported on a 0–1 scale, where values >0.5 generally correspond to proteins sharing the same fold according to SCOP/CATH classifications.

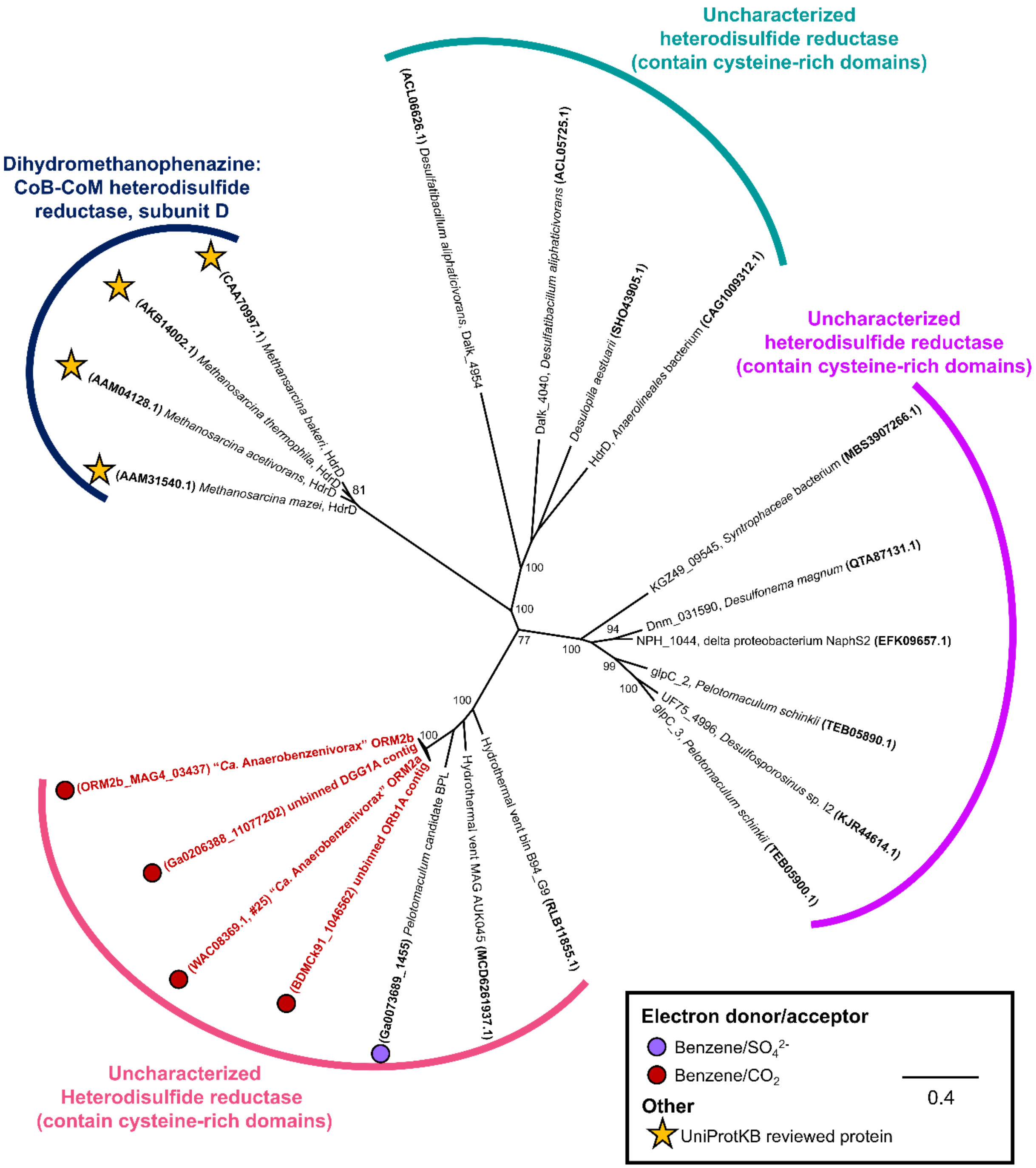

Figure S11. Maximum likelihood consensus trees showing the affiliation of Protein #25 and homologs to known and putative heterodisulfide reductase enzymes. Two proteins that mapped to unbinned OR contigs are also shown, as they share close homology (95-99% identity) with Protein #25. Proteins marked with stars have some experimental evidence of protein function and have been reviewed by UniProtKB. Bootstrap values <70% are not shown. Multiple sequence alignment statistics for most proteins are available in Table S14.

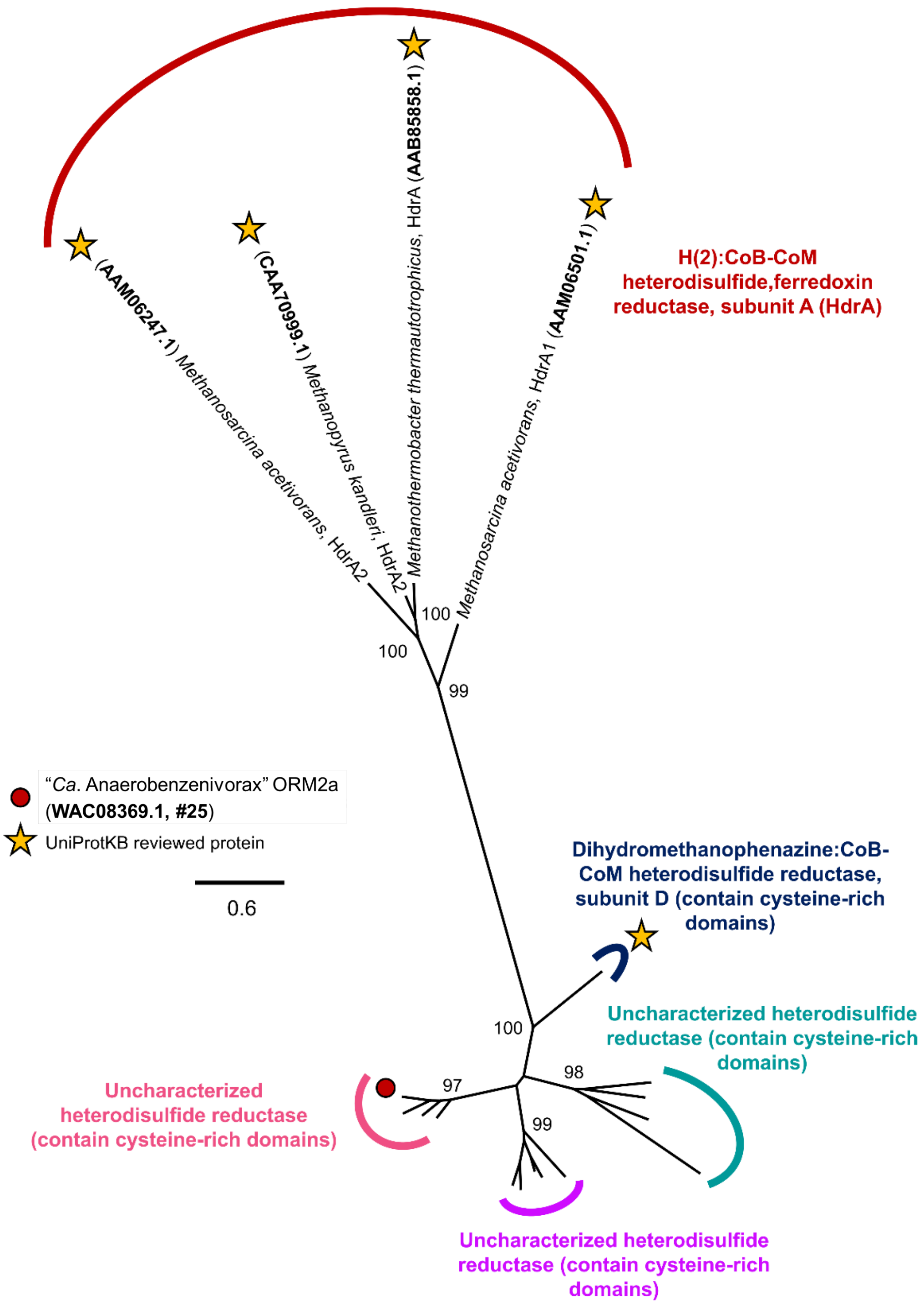

Figure S12. Maximum likelihood consensus trees of known and putative heterodisulfide reductase enzymes. This tree contains the same sequences and clustering pattern as Figure S9 but includes four HdrA sequences from methanogenic archaea. Proteins marked with stars have some experimental evidence of protein function and have been reviewed by UniProtKB. Bootstrap values < 70% are not shown.

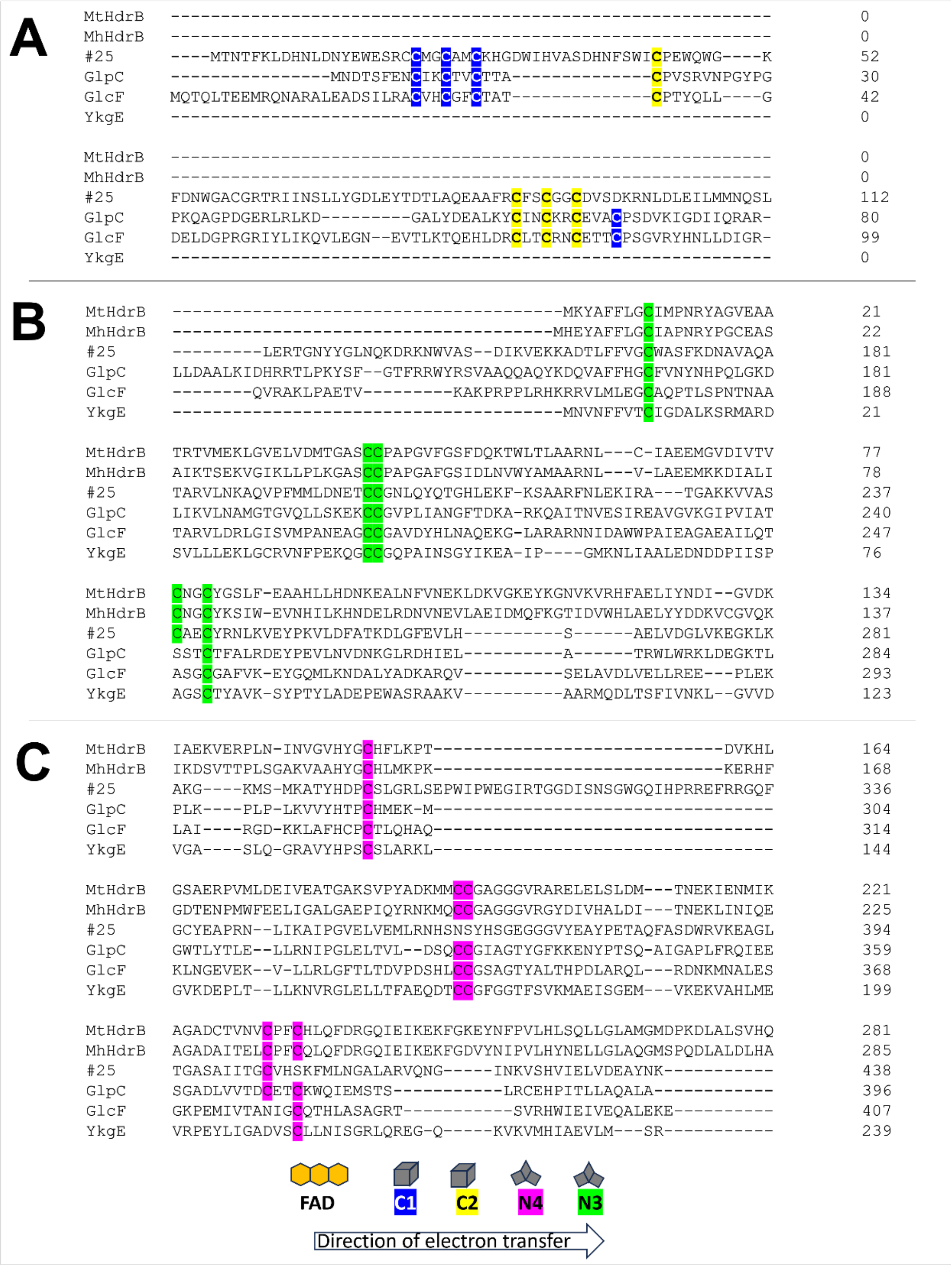
A
MtHdrB -- 0
MhHdrB -- 0
#25 ----MTNTFKLDHNLDNYEWESRCCMGCAMCKHGDWIHVASDHNFSWICPEWQWG----K 52
GlpC ----------------MNDTSFENCIKCTVCTTA--------------CPVSRVNPGYPG 30
GlcF MQTQLTEEMRQNARALEADSILRACVHCGFCTAT--------------CPTYQLL----G 42
YkgE -- 0
MtHdrB -- 0
MhHdrB -- 0
#25 FDNWGACGRTRIINSLLYGDLEYTDTLAQEAAFRCFSCGGCDVSDKRNLDLEILMMNQSL 112
GlpC PKQAGPDGERLRLKD---------GALYDEALKYCINCKRCEVACPSDVKIGDIIQRAR- 80
GlcF DELDGPRGRIYLIKQVLEGN--EVTLKTQEHLDRCLTCRNCETTCPSGVRYHNLLDIGR- 99
YkgE -- 0
B
MtHdrB ---------------------------------------MKYAFFLGCIMPNRYAGVEAA 21
MhHdrB --------------------------------------MHEYAFFLGCIAPNRYPGCEAS 22
#25 ---------LERTGNYYGLNQKDRKNWVAS--DIKVEKKADTLFFVGCWASFKDNAVAQA 181
GlpC LLDAALKIDHRRTLPKYSF--GTFRRWYRSVAAQQAQYKDQVAFFHGCFVNYNHPQLGKD 181
GlcF --------QVRAKLPAETV---------KAKPRPPLRHKRRVLMLEGCAQPTLSPNTNAA 188
YkgE ---------------------------------------MNVNFFVTCIGDALKSRMARD 21
MtHdrB TRTVMEKLGVELVDMTGASCCPAPGVFGSFDQKTWLTLAARNL---C-IAEEMGVDIVTV 77
MhHdrB AIKTSEKVGIKLLPLKGASCCPAPGAFGSIDLNVWYAMAARNL---V-LAEEMKKDIALI 78
#25 TARVLNKAQVPFMMLDNETCCGNLQYQTGHLEKF-KSAARFNLEKIRA---TGAKKVVAS 237
GlpC LIKVLNAMGTGVQLLSKEKCCGVPLIANGFTDKA-RKQAITNVESIREAVGVKGIPVIAT 240
GlcF TARVLDRLGISVMPANEAGCCGAVDYHLNAQEKG-LARARNNIDAWWPAIEAGAEAILQT 247
YkgE SVLLLEKLGCRVNFPEKQGCCGQPAINSGYIKEA-IP----GMKNLIAALEDNDDPIISP 76
MtHdrB CNGCYGSLF-EAAHLLHDNKEALNFVNEKLDKVGKEYKGNVKVRHFAELIYNDI--GVDK 134
MhHdrB CNGCYKSIW-EVNHILKHNDELRDNVNEVLAEIDMQFKGTIDVWHLAELYYDDKVCGVQK 137
#25 CAECYRNLKVEYPKVLDFATKDLGFEVLH----------S------AELVDGLVKEGKLK 281
GlpC SSTCTFALRDEYPEVLNVDNKGLRDHIEL----------A------TRWLWRKLDEGKTL 284
GlcF ASGCGAFVK-EYGQMLKNDALYADKARQV----------SELAVDLVELLREE---PLEK 293
YkgE AGSCTYAVK-SYPTYLADEPEWASRAAKV----------AARMQDLTSFIVNKL--GVVD 123
C
MtHdrB IAEKVERPLN-INVGVHYGCHFLKPT-----------------------------DVKHL 164
MhHdrB IKDSVTTPLSGAKVAAHYGCHLMKPK-----------------------------KERHF 168
#25 AKG----KMS-MKATYHDPCSLGRLSEPWIPWEGIRTGGDISNSGWGQIHPRREFRRGQF 336
GlpC PLK----PLP-LKVVYHTPCHMEK-M---------------------------------- 304
GlcF LAI----RGD-KKLAFHCPCTLQHAQ---------------------------------- 314
YkgE VGA----SLQ-GRAVYHPSCSLARKL---------------------------------- 144
MtHdrB GSAERPVMLDEIVEATGAKSVPYADKMMCCGAGGGVRARELELSLDM---TNEKIENMIK 221
MhHdrB GDTENPMWFEELIGALGAEPIQYRNKMQCCGAGGGVRGYDIVHALDI---TNEKLINIQE 225
#25 GCYEAPRN--LIKAIPGVELVEMLRNHSNSYHSGEGGGVYEAYPETAQFASDWRVKEAGL 394
GlpC GWTLYTLE--LLRNIPGLELTVL--DSQCCGIAGTYGFKKENYPTSQ-AIGAPLFRQIEE 359
GlcF KLNGEVEK--V-LLRLGFTLTDVPDSHLCCGSAGTYALTHPDLARQL---RDNKMNALES 368
YkgE GVKDEPLT--LLKNVRGLELLTFAEQDTCCGFGGTFSVKMAEISGEM---VKEKVAHLME 199
MtHdrB AGADCTVNVCPFCHLQFDRGQIEIKEKFGKEYNFPVLHLSQLLGLAMGMDPKDLALSVHQ 281
MhHdrB AGADAITELCPFCQLQFDRGQIEIKEKFGDVYNIPVLHYNELLGLAQGMSPQDLALDLHA 285
#25 TGASAIITGCVHSKFMLNGALARVQNG-----INKVSHVIELVDEAYNK----------- 438
GlpC SGADLVVTDCETCKWQIEMSTS-----------LRCEHPITLLAQALA------------ 396
GlcF GKPEMIVTANIGCQTHLASAGRT-----------SVRHWIEIVEQALEKE---------- 407
YkgE VRPEYLIGADVSCLLNISGRLQREG-Q-----KVKVMHIAEVLM---SR----------- 239
FAD
C1
C2
N4
N3
Direction of electron transfer

Figure S13 (above). Multiple sequence alignment of cysteine-rich motifs in Protein #25 and selected iron-sulfur homologs from methanogens and *Escherichia coli*, specifically HdrB subunits from *Methanothermococcus thermolithotrophicus* (MtHdrB, PDB: 5ODQ) and *Methanospirillum hungatei* (MhHdrB, PDB: 7BKE), *E. coli* glycerol-3-phosphate dehydrogenase (GlpC, UniProt: P0A996), *E. coli* glycolate dehydrogenase (GlcF, P52074), and *E. coli* L-lactate utilization protein (YkgE, P77252). Shown in top panel (A), the N-terminal regions of GlpC and GlcF are predicted to bind two cubane [4Fe-4S] clusters via an 8-Cys motif (highlighted blue and yellow), of which 7-Cys are conserved in Protein #25. In the center panel (B), MtHrdB and MhHrdB bind the first non-cubane [4Fe-4S] cluster via a 5-Cys motif (green), of which 4- or 5-Cys are conserved in the bacterial proteins. In the bottom panel (C), MtHrdB and MhHrdB bind the second non-cubane [4Fe-4S] cluster via a 5-Cys motif (pink), of which 4- or 5-Cys are conserved in the *E. coli* proteins, and only 2-Cys are conserved in Protein #25. The schematic shows the predicted electron flow from FAD to the cubane and non-cubane [4Fe-4S] clusters.

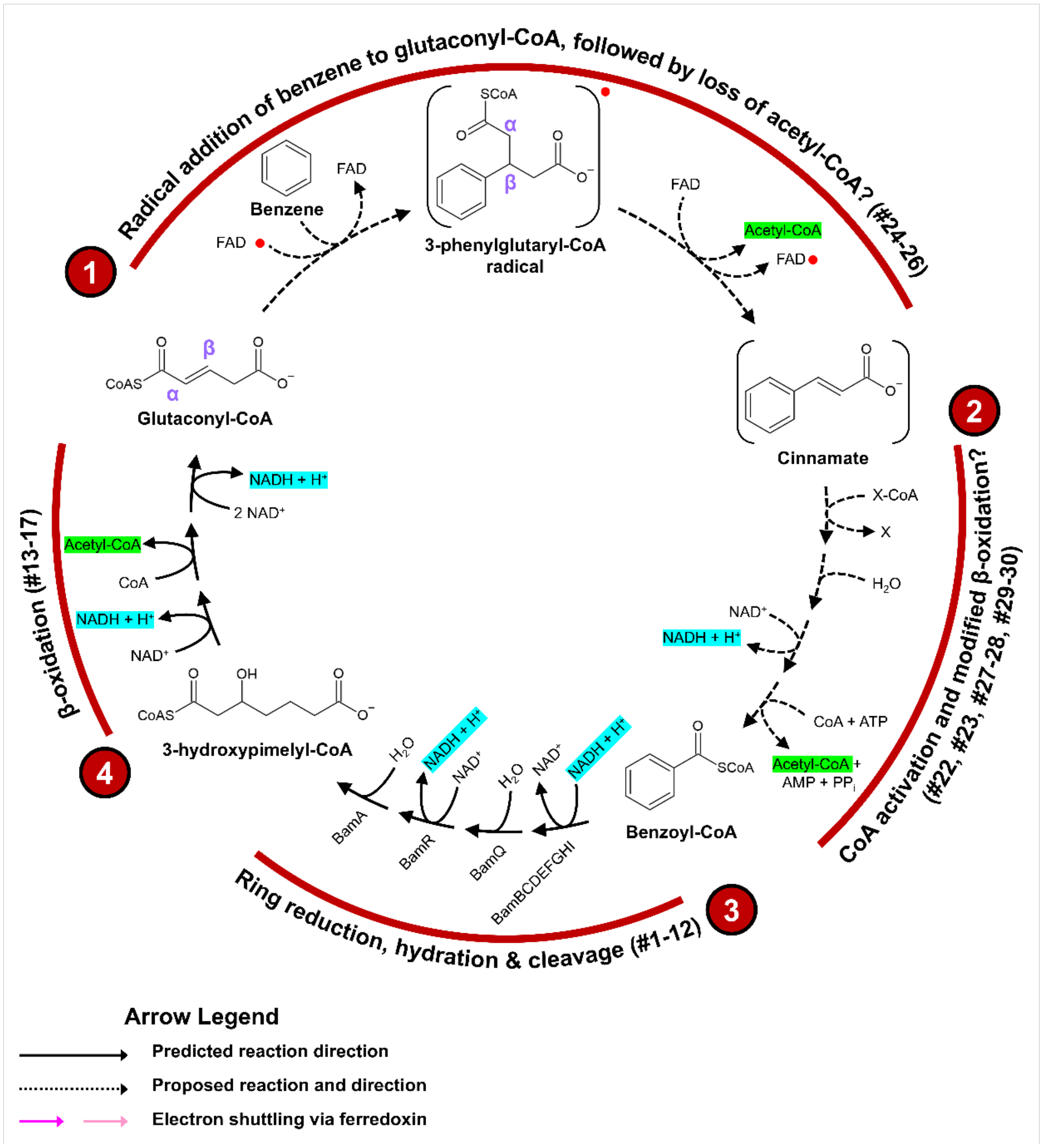

Figure S14. Conceptual model of anaerobic benzene degradation by ORM2a. We speculate that a flavin-mediated radical reaction might be used to add benzene to glutaconyl-CoA. Radicals are denoted by red circles; hypothetical intermediates are indicated by closed brackets. This closed-loop model suggests that glutaconyl-CoA does not undergo further β-oxidation steps yielding acetyl-CoA. Stoichiometric production of hydrogen equivalents (blue) and acetyl-CoA (green) is highlighed. Electron balances for each reaction step are shown in Table S17.

REFERENCES

1. Nales M, Butler BJ, Edwards EA. 1998. Anaerobic benzene biodegradation: A microcosm survey. Bioremediat J 2:125-144.
2. Ulrich AC, Edwards EA. 2003. Physiological and molecular characterization of anaerobic benzene-degrading mixed cultures. Environ Microbiol 5:92-102.
3. Guo S, Toth CRA, Luo F, Chen X, Xiao J, Edwards EA. 2022. Transient oxygen exposure causes profound and lasting changes to a benzene-degrading methanogenic community. Environ Sci Technol 56:13036-13045.
4. Luo F, Devine CE, Edwards EA. 2016. Cultivating microbial dark matter in benzene-degrading methanogenic consortia. Environ Microbiol 18:2923-2936.
5. Chen X, Molenda O, Brown CT, Toth CRA, Guo S, Luo F, Howe J, Nesbø C, He C, Montabana EA, Cate JHD, Banfield JF, Edwards EA. 2023. "*Candidatus* Nealsonbacteria" are likely biomass recycling ectosymbionts of methanogenic archaea in a stable benzene-degrading enrichment culture. Appl Environ Microbiol 89:e00025-23.
6. Devine CE. 2013. Identification of key organisms, genes and pathways in benzene-degrading methanogenic cultures. PhD thesis. University of Toronto, Toronto, Canada.
7. Mancini SA, Devine CE, Elsner M, Nandi ME, Ulrich AC, Edwards EA, Sherwood Lollar B. 2008. Isotopic evidence suggests different initial reaction mechanisms for anaerobic benzene biodegradation. Environ Sci Technol 42:8290-8296.
8. Toth CRA, Molenda O, Nesbø C, Luo F, Devine C, Guo S, Chen X, Edwards EA. 2023. Metagenomic and genomic sequences from a methanogenic benzene-degrading consortium. Microbiol Resour Announc 12:e01342-22.
9. Toth CRA, Luo F, Bawa N, Webb J, Guo S, Dworatzek S, Edwards EA. 2021. Anaerobic benzene biodegradation linked to the growth of highly specific bacterial clades. Environ Sci Technol 55:7970-7980.
10. Chen X, Toth CRA, Guo S, Luo F, Howe J, Nesbø CL, Edwards EA. 2025. Visualization of syntrophic benzene-fermenting *Desulfobacterota* ORM2 in a methanogenic enrichment culture using fluorescence in situ hybridization. Environ Sci Technol 59:591-602.
11. Luo F. 2016. Characterization of the microbial community composition and benzene activation mechanisms in anaerobic benzene-degrading enrichment cultures. PhD thesis. University of Toronto, Toronto, Canada.
12. Simpson JT, Wong K, Jackman SD, Schein JE, Jones SJ, Birol I. 2009. ABySS: a parallel assembler for short read sequence data. Genome Res 19:1117-1123.
13. Toth CRA, Molenda O, Nesbo C, Luo F, Devine C, Guo S, Chen X, Edwards EA. 2022. FASTA files and relevant statistics for draft MAGs generated from a methanogenic benzene-degrading enrichment culture. figshare Dataset, https://doi.org/10.6084/m9.figshare.21663302.
14. Chaumeil PA, Mussig AJ, Hugenholtz P, Parks DH. 2019. GTDB-Tk: a toolkit to classify genomes with the Genome Taxonomy Database. Bioinformatics 36:1925-1927.
15. Parks DH, Chuvochina M, Rinke C, Mussig AJ, Chaumeil P-A, Hugenholtz P. 2021. GTDB: an ongoing census of bacterial and archaeal diversity through a phylogenetically consistent, rank normalized and complete genome-based taxonomy. Nucleic Acids Res 50:D785-D794.
16. Brettin T, Davis JJ, Disz T, Edwards RA, Gerdes S, Olsen GJ, Olson R, Overbeek R, Parrello B, Pusch GD, Shukla M, Thomason JA, Stevens R, Vonstein V, Wattam AR, Xia

F. 2015. *RASTtk*: A modular and extensible implementation of the RAST algorithm for building custom annotation pipelines and annotating batches of genomes. Sci Rep 5:8365.
17. Kanehisa M, Sato Y, Morishima K. 2016. BlastKOALA and GhostKOALA: KEGG tools for functional characterization of genome and metagenome sequences. J Mol Biol 428:726-731.
18. Kanehisa M, Sato Y. 2020. KEGG Mapper for inferring cellular functions from protein sequences. Protein Sci 29:28-35.
19. Kanehisa M, Sato Y, Kawashima M. 2022. KEGG mapping tools for uncovering hidden features in biological data. Protein Sci 31:47-53.
20. Krzywinski M, Schein J, Birol I, Connors J, Gascoyne R, Horsman D, Jones SJ, Marra MA. 2009. Circos: An information aesthetic for comparative genomics. Genome Res 19:1639-1645.
21. Dong M-J, Luo H, Gao F. 2022. Ori-Finder 2022: a comprehensive web server for prediction and analysis of bacterial replication origins. Genom Proteom Bioinform 20:1207-1213.
22. Bertelli C, Laird MR, Williams KP, Simon Fraser University Research Computing Group, Lau BY, Hoad G, Winsor GL, Brinkman FSL. 2017. IslandViewer 4: expanded prediction of genomic islands for larger-scale datasets. Nucleic Acids Res 45:W30-W35.
23. Arndt D, Grant J, Marcu A, Sajed T, Pon A, Liang Y, Wishart DS. 2016. PHASTER: a better, faster version of the PHAST phage search tool. Nucleic Acids Res 44:W16-W21.
24. Siguier P, Perochon J, Lestrade L, Mahillon J, Chandler M. 2006. ISfinder: the reference centre for bacterial insertion sequences. Nucleic Acids Res 34:D32-D36.
25. Taboada B, Estrada K, Ciria R, Merino E. 2018. Operon-mapper: a web server for precise operon identification in bacterial and archaeal genomes. Bioinformatics 34:4118-4120.
26. Wiśniewski JR, Zougman A, Nagaraj N, Mann M. 2009. Universal sample preparation method for proteome analysis. Nat Methods 6:359-362.
27. Shevchenko A, Tomas H, Havlis J, Olsen JV, Mann M. 2006. In-gel digestion for mass spectrometric characterization of proteins and proteomes. Nat Protoc 1:2856-2860.
28. Tang S, Chan WWM, Fletcher KE, Seifert J, Liang X, Löffler FE, Edwards EA, Adrian L. 2013. Functional characterization of reductive dehalogenases by using blue native polyacrylamide gel electrophoresis. Appl Environ Microbiol 79:974-981.
29. Kessner D, Chambers M, Burke R, Agus D, Mallick P. 2008. ProteoWizard: open source software for rapid proteomics tools development. Bioinformatics 24:2534-2536.
30. Keller A, Nesvizhskii AI, Kolker E, Aebersold R. 2002. Empirical statistical model to estimate the accuracy of peptide identifiations made by MS/MS and database search. Anal Chem 74:5383-5392.
31. Nesvizhskii AI, Keller A, Kolker E, Aebersold R. 2003. A statistical model for identifying proteins by tandem mass spectrometry. Anal Chem 75:4646-4658.
32. Leung WHP, Chi Tam W, Chang BCH, Halgamuge SK. 2003. Effects of search pattern variations in motif discovery algorithm: MotifFinder. IFAC Proc Vol 36:501-506.
33. Jumper J, Evans R, Pritzel A, Green T, Figurnov M, Ronneberger O, Tunyasuvunakool K, Bates R, Žídek A, Potapenko A, Bridgland A, Meyer C, Kohl SAA, Ballard AJ, Cowie A, Romera-Paredes B, Nikolov S, Jain R, Adler J, Back T, Petersen S, Reiman D, Clancy E, Zielinski M, Steinegger M, Pacholska M, Berghammer T, Bodenstein S, Silver D, Vinyals O, Senior AW, Kavukcuoglu K, Kohli P, Hassabis D. 2021. Highly accurate protein structure prediction with AlphaFold. Nature 596:583-589.

34. Mirdita M, Schütze K, Moriwaki Y, Ovchinnikov S, Steinegger M. 2022. ColabFold: making protein folding accessible to all. Nat Methods 19:679-682.
35. Holm L. 2022. Dali server: structural unification of protein families. Nucleic Acids Res 50:W210-W215.
36. Gligorijević V, Renfrew PD, Kosciolek T, Leman JK, Berenberg D, Vatanen T, Chandler C, Taylor BC, Fisk IM, Vlamakis H, Xavier RJ, Knight R, Cho K, Bonneau R. 2021. Structure-based protein function prediction using graph convolutional networks. Nat Commun 12:3168.
37. Abramson J, Adler J, Dunger J, al. e. 2024. Accurate structure prediction of biomolecular interactions with AlphaFold 3. Nature 630:493-500.
38. Stamatakis A. 2014. RAxML version 8: a tool for phylogenetic analysis and post-analysis of large phylogenies. Bioinformatics 30:1312-1313.
39. Kearse M, Moir R, Wilson A, Stones-Havas S, Cheung M, Sturrock S, Buxton S, Cooper A, Markowitz S, Duran C, Thierer T, Ashton B, Meintjes P, Drummond A. 2012. Geneious Basic: An integrated and extendable desktop software platform for the organization and analysis of sequence data. Bioinformatics 28:1647-1649.
40. Waman VP, Bordin N, Lau A, Kandathil S, Wells J, Miller D, Velankar S, Jones DT, Sillitoe I, Orengo C. 2024. CATH v4.4: major expansion of CATH by experimental and predicted structural data. Nucleic Acids Res 53:D348–D355.
41. Kilinc M, Jia K, Jernigan RL. 2023. Improved global protein homolog detection with major gains in function identification. PNAS 120:e2211823120.
42. Lee MD. 2019. GToTree: a user-friendly workflow for phylogenomics. Bioinformatics 35:4162-4164.
43. Edgar RC. 2004. MUSCLE: multiple sequence alignment with high accuracy and high throughput. Nucleic Acids Res 32:1792-1797.
44. Nguyen L-T, Schmidt HA, von Haeseler A, Minh BQ. 2014. IQ-TREE: a fast and effective stochastic algorithm for estimating maximum-likelihood phylogenies. Mol Biol Evol 32:268-274.
45. Elshahed MS, McInerney MJ. 2001. Benzoate fermentation by the anaerobic bacterium *Syntrophus aciditrophicus* in the absence of hydrogen-using microorganisms. Appl Environ Microbiol 67:5520-5525.
46. McInerney MJ, Rohlin L, Moutakki H, Kim U, Krupp RS, Rios-Hernandez L, Sieber J, Struchtemeyer CG, Bhattacharyya A, Campbell JW, Gunsalus RP. 2007. The genome of *Syntrophus aciditrophicus*: Life at the thermodynamic limit of microbial growth. PNAS 104:7600-7605.
47. Mancini SA, Ulrich AC, Lacrampe-Couloume G, Sleep B, Edwards EA, Lollar BS. 2003. Carbon and hydrogen isotopic fractionation during anaerobic biodegradation of benzene. Appl Environ Microbiol 69:191-198.
48. Ulrich AC, Beller HR, Edwards EA. 2005. Metabolites detected during biodegradation of $^{13}C_6$-benzene in nitrate-reducing and methanogenic enrichment cultures. Environ Sci Technol 39:6681-6691.
49. Mukherjee A, Zhang Y. 2009. MM-align: a quick algorithm for aligning multiple-chain protein complex structures using iterative dynamic programming. Nucleic Acids Res 37:e83.
